# Stability of Zr-Based UiO-66 Metal–Organic Frameworks in Basic Solutions

**DOI:** 10.3390/nano14010110

**Published:** 2024-01-02

**Authors:** Jun Yeong Kim, Jiwon Kang, Seungheon Cha, Haein Kim, Dopil Kim, Houng Kang, Isaac Choi, Min Kim

**Affiliations:** 1 Department of Chemistry, Chungbuk National University, Cheongju 28644, Republic of Korea; junyeongkim1111@gmail.com (J.Y.K.); kangjiwon1001@gmail.com (J.K.); chaseungheon045@gmail.com (S.C.); haeinkim9951@gmail.com (H.K.); ehvlfdl0218@gmail.com (D.K.); 2 Department of Chemistry Education, Chungbuk National University, Cheongju 28644, Republic of Korea; hkang@chungbuk.ac.kr

**Keywords:** metal–organic frameworks, zirconium, UiO-66, basic solution, stability

## Abstract

Although Zr-based metal–organic frameworks (MOFs) exhibit robust chemical and physical stability in the presence of moisture and acidic conditions, their susceptibility to nucleophilic attacks from bases poses a critical challenge to their overall stability. Herein, we systematically investigate the stability of Zr-based UiO-66 (UiO = University of Oslo) MOFs in basic solutions. The impact of 11 standard bases, including inorganic salts and organic bases, on the stability of these MOFs is examined. The destruction of the framework is confirmed through powder X-ray diffraction (PXRD) patterns, and the monitored dissolution of ligands from the framework is assessed using nuclear magnetic resonance (NMR) spectroscopy. Our key findings reveal a direct correlation between the strength and concentration of the base and the destruction of the MOFs. The summarized data provide valuable insights that can guide the practical application of Zr-based UiO-66 MOFs under basic conditions, offering essential information for their optimal utilization in various settings.

## 1. Introduction

Metal–organic frameworks (MOFs) represent a class of materials based on metal–ligand coordination, characterized by a three-dimensional structure and inherent porosity. The versatility of MOFs is underscored by the utilization of various metal salts and clusters coupled with a diverse array of coordinating ligands (e.g., carboxylate or pyridyl groups) employed in their synthesis. This diversity allows for a broad spectrum of MOF compositions, imparting distinctive chemical and physical properties to these materials [1,2,3,4,5,6].

One defining feature of MOFs is their predictable reticular chemistry, which enables the exploration of similar structures with varying ligand lengths and identical frameworks originating from identical coordinating sites but featuring different functional groups [7,8,9,10]. This aspect has been extensively investigated in the field of MOF chemistry. The porosity of MOFs has been exploited for efficient gas storage and molecular separation, leveraging sieving effects, and their unique structural characteristics have found applications in diverse fields [11,12,13,14,15,16]. The tunable pores of MOFs hold promise for selective molecular conveyance, such as drug delivery applications [17,18,19]. Furthermore, the recurring combinations of metal–ligand coordination lead to a distinctive catalytic efficacy in reactions, contribute to optical properties and have the potential for applications in energy-related processes [4,20,21]. However, the practical applications of MOFs have been hindered by their relatively low stability under moisture or acidic/basic conditions. Addressing these stability challenges is crucial for unlocking the full potential of these sophisticated materials for real-world applications in various industries as well as in daily life [22,23,24,25,26,27]. The stability of MOFs has been investigated through theoretical and experimental approaches [28,29,30,31,32,33,34,35,36], and the effects of buffers, amino acids, and cell media on MOFs have been extensively examined [37,38,39].

For this reason, diverse strategies for the increase in their stability have been employed during the preparation steps [23,40,41]. Notably, the nature of the metal and ligand, as elucidated by the hard–soft acid–base (HSAB) theory, is pivotal [42,43]. Additionally, introducing hydrophobic characteristics and safeguarding frameworks have been the subject of extensive investigation [44,45,46]. For instance, the choice of metal ions and ligands significantly influences stability. Applying the HSAB theory, MOFs featuring hard carboxylate ligands, particularly those anchored to zirconium (Zr, a hard metal ion), exhibit superior stability against water compared to MOFs that incorporate softer metal ions such as zinc [47,48]. Consequently, Zr-based MOFs have emerged as a focal point in a plethora of research, demonstrating enhanced stability and versatility for various applications, including industrial uses [49,50,51,52,53,54].

The pioneering work on Zr-based MOFs is exemplified by the UiO series, employing benzene-1,4-dicarboxylate (BDC) ligands, as reported by Lillerud et al. [55]. Subsequent studies have explored manipulating the connectivity of secondary building units and the coordination of ligands, transitioning from dicarboxylic acids to tricarboxylic and tetracarboxylic acids. The exceptional stability of Zr-based MOFs in water and acidic conditions, coupled with their diverse structural possibilities and relative ease of accessibility, position them as representative MOFs across various applications spanning diverse research fields [49,50,51,52,53,54].

Despite the numerous advantages of Zr-based MOFs, the persistent challenge of low stability under basic conditions remains unresolved. Recurrent nucleophilic attacks by hydroxide ions (OH^−^) on Zr–carboxylate coordination bonds lead to the dissolution of Zr-based MOFs [56]. This instability is intricately linked to structural issues and the properties of the pore environments. Notably, Zr-based UiO-66 MOFs are commonly reported to be unstable under basic conditions [56], with a limitation level at pH 12 [41]. This inherent instability poses a significant obstacle, especially in the realm of catalytic applications in organic reactions, where bases are often used as essential additives. For instance, new bond-forming cross-coupling reactions, such as the Suzuki–Miyaura reaction and Buchwald–Hartwig amination, require a wide range of bases [57], translating the fact that the low stability of Zr-MOFs under basic conditions restricts their widespread use in applications in organic syntheses [58,59,60,61,62].

To address these concerns, our work focuses on the systematic study of Zr-MOFs in basic solutions to determine their stability. The UiO-66 system, which is based on the simplest BDC ligand, was chosen for investigation because of its ease of accessibility and high chemical stability. Our investigations encompass various inorganic and organic bases, and the insights gained from this study offer valuable information for the practical application of Zr-MOFs, particularly in scenarios involving basic conditions and catalytic applications.

## 2. Results and Discussion

### 2.1. Preparation of Zr-Based UiO-66 MOFs

UiO-66, the primary Zr-based MOF investigated in this study, was synthesized following Farha’s solvothermal protocol [63]. The synthesis involved ZrCl^4^ and H^2^BDC (benzene-1,4-dicarboxylic acid or terephthalic acid) with formic acid modulators. Detail procedures are provided in the Supplementary Materials (p. S2). Acid modulators are commonly employed in the solvothermal synthesis of MOFs to regulate crystallization speed and enhance reproducibility. However, using acid modulators introduced structural defects into the pristine UiO-66 MOF, a characteristic arising from the consistent protocol employed [64,65,66].

**Figure 1:**
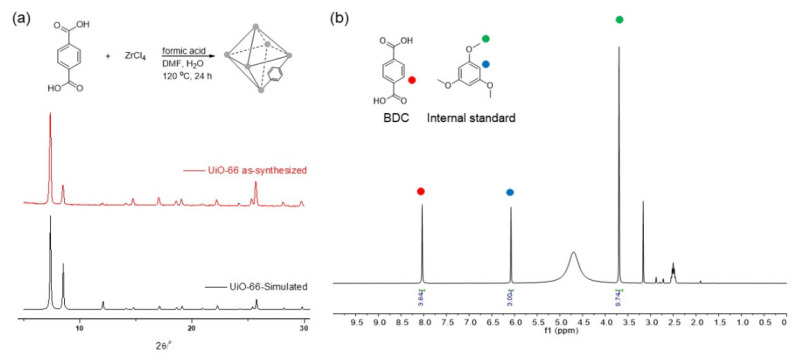
(**a**) Preparation of UiO-66 MOF and PXRD and (**b**) NMR data of pristine UiO-66 MOF (the digested sample) before base treatments.

The crystallinity and stability of the synthesized MOFs were initially validated using powder X-ray diffraction (PXRD) pattern analysis. The PXRD patterns of the as-synthesized MOFs were compared with a simulated pattern derived from a reported structure (Figure 1). Any deviations, such as peak broadening in UiO-66, indicated framework destruction. The dissolution of ligands in the MOFs under basic conditions was monitored through physical mass measurements after basic treatment to further assess the stability. Additionally, ^1^H nuclear magnetic resonance (NMR) spectroscopy data acquired after acid digestion provided insight into ligand dissolution. An internal standard method was employed to quantify the BDC ligands within the framework, utilizing 1,3,5-trimethoxy benzene as the NMR internal standard. Detail protocols for PXRD and NMR are provided in the Supplementary Materials (p. S2). Before subjecting UiO-66 to the basic treatment, the pristine MOFs exhibited 91% of the expected BDC ligands compared to the ideal structure (Figure 1). This 9% discrepancy could be attributed to structural defects resulting from the use of formic acid modulators. Further comparisons of the remaining BDC ligand amounts (by ^1^H NMR) post-basic treatments shed light on the variations in stability. Notably, the introduction of additional functional groups to the ligand was not considered in this analysis. This omission was due to the potential impact of functional groups (e.g., nitro or tetramethyl groups) on MOF stability [41,67], which could introduce confounding variables into the study.

### 2.2. Effects of Inorganic Base Solutions on UiO-66 MOF

To assess the stability of the UiO-66 MOF under basic conditions, we selected representative potassium salts, including KOH, K_3_PO_4_, K_2_CO_3_, KHCO_3_, KOAc, and K_2_SO_4_. These inorganic bases were chosen to ensure a comprehensive evaluation while excluding cationic effects on MOF stability. The p*K*_b_ values of these bases ranged from −1.7 to 12 (Table 1) [68,69,70]. Initial stability tests involved incubating 30 mg of UiO-66 MOF in each 0.1 M aqueous basic solution for 1 h, followed by recovery through centrifugation and PXRD analysis after washing with pure methanol to remove trapped water molecules.

**Figure 2:**
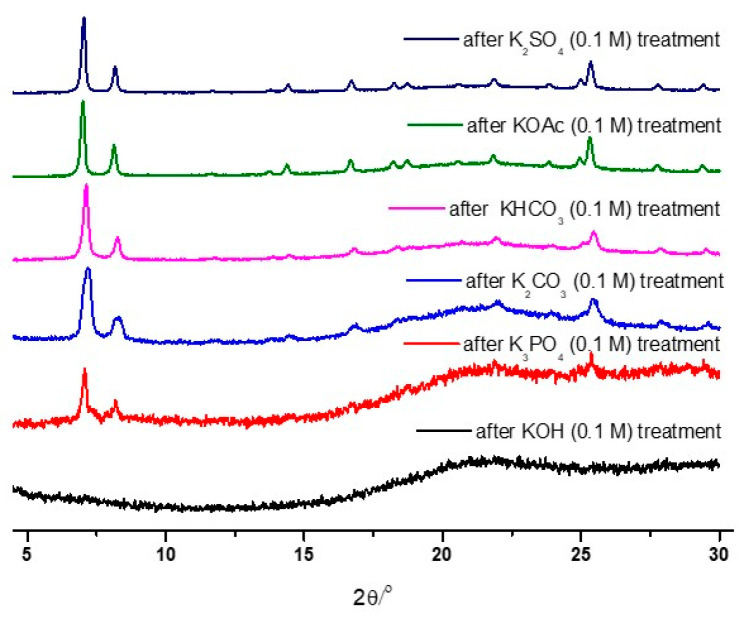
PXRD patterns of the recovered UiO-66 samples after the 0.1 M basic solution treatment.

As illustrated in Figure 2, the strongest base, KOH, led to the total destruction of the UiO-66 structure within 1 h. Conversely, weak bases, such as KHCO_3_, KOAc, and K_2_SO_4_, generally retained their PXRD patterns after 0.1 M of the solution treatment. Therefore, detailed analyses were performed with K_3_PO_4_, KHCO_3_, and KOAc by varying the concentration and treatment time.

**Figure 3:**
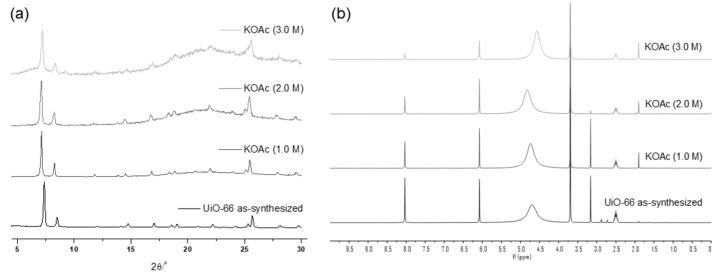
(**a**) PXRD patterns and (**b**) NMR spectral changes after treatment with the KOAc solution for 1 h.

The weak base, KOAc, demonstrated good tolerance for the UiO-66 MOF, with no changes in the PXRD patterns observed until treatment with a 1.0 M KOAc solution for 1 d. Structural decomposition commenced at a 2.0 M solution, and substantial destruction occurred with a 3.0 M KOAc solution for 1 h, leaving only 31% remaining BDC ligands in the solid-state MOF (Figure 3) compared to the ideal UiO-66 MOF. Since the starting MOF had 91% of BDC ligands in their structure (and 9% of the defect), 60% of the ligand was removed from the framework during base treatments.

**Figure 4:**
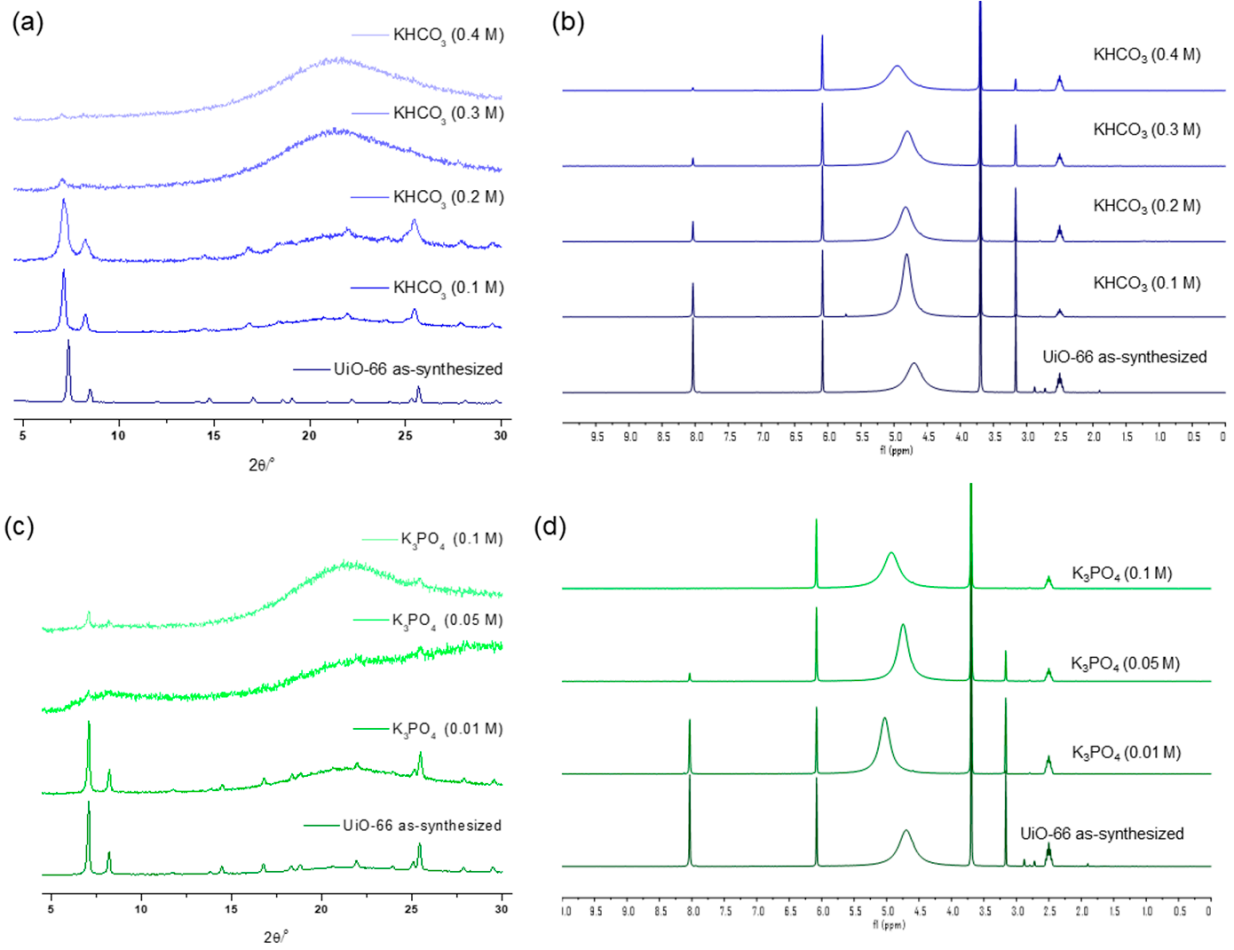
(**a**) PXRD patterns and (**b**) NMR spectral changes after KHCO_3_ solution treatment for 1 h. (**c**) PXRD patterns and (**d**) NMR spectral changes after K_3_PO_4_ solution treatment for 1 h.

Specific concentration effects were examined using medium-strength bases, KHCO_3_ and K_3_PO_4_. KHCO_3_ exhibited a PXRD peak that broadened at 0.2 M of the solution treatment for 1 h, with complete framework destruction confirmed at 0.3 M of solution incubation for 1 h, leaving less than 10% of the remaining ligands in the solid state (which meant an 81% loss from pristine MOF, Figure 4). Between K_3_PO_4_ and KHCO_3_, the stronger base, K_3_PO_4_, annihilated the UiO-66 structure with a 0.05 M solution within 1 h, with almost no remaining BDC ligand, as confirmed via the ^1^H NMR analysis after acid digestion (Figure 4).

**Figure 5:**
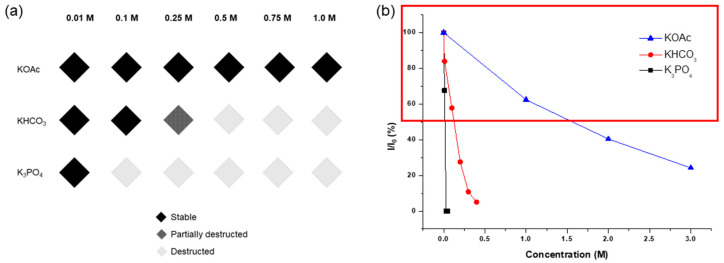
(**a**) Concentration limits for inorganic base treatments of UiO-66 and (**b**) the remaining BDC ligands in the recovered UiO-66 after each base treatment.

The concentration limits and remaining ligands are summarized in Figure 5. Notably, KOH exterminated all MOF solid materials with a 0.1 M solution for 1 h, while KOAc exhibited tolerance even with a 1.0 M solution for 1 d. K_3_PO_4_ led to over half the destruction with a 0.05 M solution for 1 h, and KHCO_3_ resulted in similar framework destruction with a 0.2 M solution for 1 h of incubation. Consequently, it was confirmed that the acetate series is a promising inorganic base additive for using Zr-MOFs under basic conditions.

### 2.3. Effects of Organic Base Solutions on UiO-66 MOF

**Figure 6:**
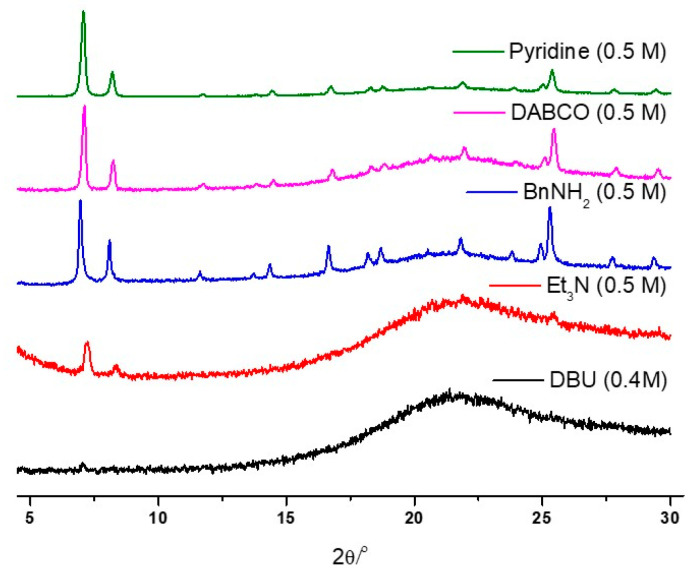
PXRD patterns of the recovered UiO-66 samples after the organic basic solution treatment.

To assess the stability of UiO-66 MOFs under basic conditions, standard organic bases were examined, including DBU (1,8-diazabicyclo[5.4.0]undec-7-ene), Et_3_N (triethylamine), BnNH_2_ (benzylamine), DABCO (1,4-diazabicyclo[2.2.2]octane), and pyridine. Initial stability tests were conducted using 0.5 M of aqueous solutions, and the p*K*_b_ values of these organic bases ranged from 0.5 to 8.77 (Table 2) [71]. The strong bases, DBU and Et_3_N, resulted in the complete destruction of the UiO-66 frameworks within 1 h of the 0.5 M concentration test (Figure 6). Furthermore, the concentration limits were explored using DBU, DABCO, and pyridine.

**Figure 7:**
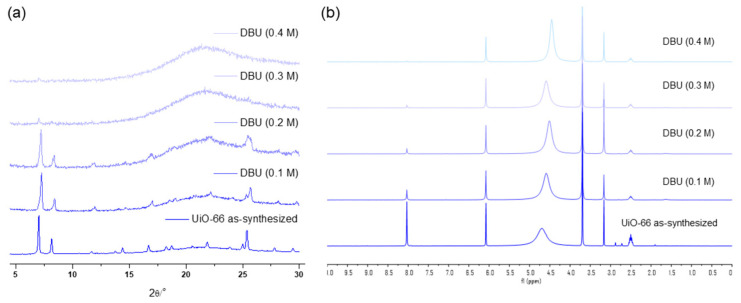

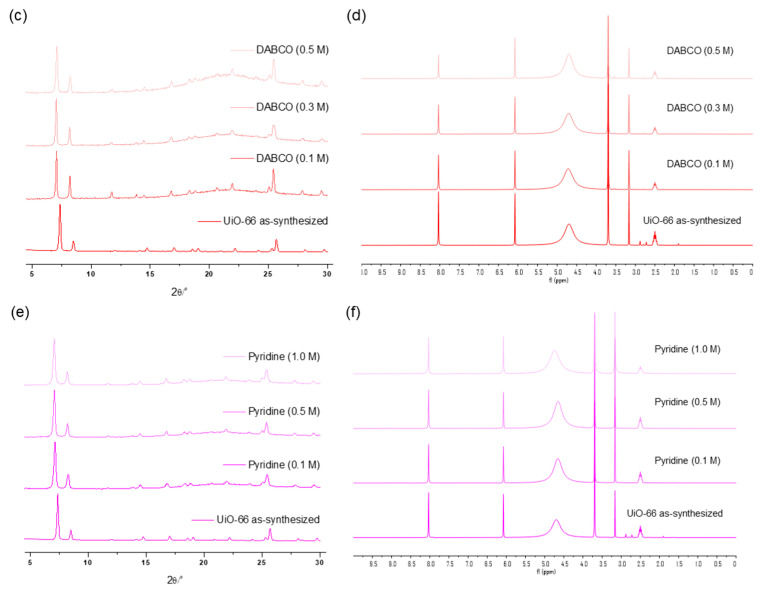
(**a**) PXRD patterns and (**b**) NMR spectral changes after DBU solution treatment for 1 h. (**c**) PXRD patterns and (**d**) NMR spectral changes after DABCO solution treatment for 1 h. (**e**) PXRD patterns and (**f**) NMR spectral changes after pyridine solution treatment for 1 h.

The concentration variation revealed that 0.3 M was the limit for a one-hour treatment of UiO-66 MOFs with the DBU solution. Although all the primary peaks in the PXRD of UiO-66 were retained with a 0.2 M solution treatment, 0.3 M of the DBU solution removed all the primary peaks from the PXRD patterns (Figure 7). Meanwhile, the portion of the remaining BDC ligand in the solid state was drastically decreased in the 0.3 M DBU solution treatment (Figure 7). In contrast, DABCO, which is often used as a nitrogen donor ligand for MOF synthesis, showed good compatibility with the Zr-MOFs. Although some peak broadenings were observed, all primary peaks of the UiO-66 frameworks were completely retained. In addition, ligand dissolution was much lower than that in the DBU treatment (Figure 7). Notably, higher concentration tests (>0.5 M) were unsuccessful because of the low solubility of DBU and DABCO in water. Finally, in the case of the weakest pyridine, the PXRD pattern of the UiO-66 MOF retained its sharpness until the 1.0 M treatment, and the BDC ligands remained in their solid state (Figure 7).

**Figure 8:**
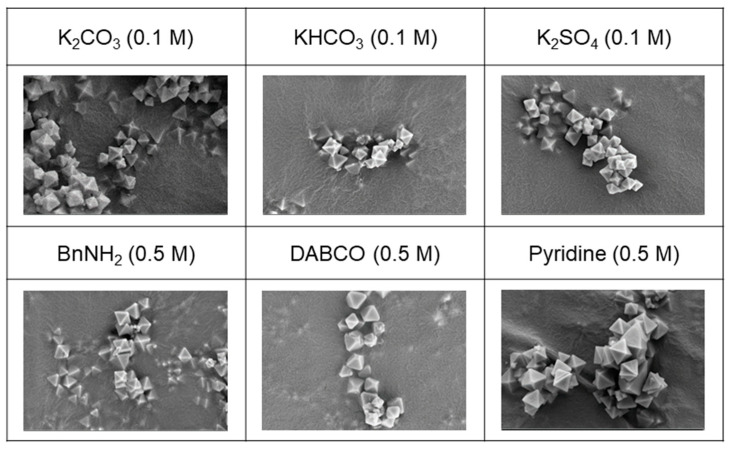
SEM images (×20,000) of recovered UiO-66 after base treatments.

Six samples, each treated with 0.1 M of K_2_CO_3_, KHCO_3_, and K_2_SO_4_ as inorganic bases and 0.5 M of BnNH_2_, DABCO, and pyridine as organic bases, were carefully chosen for in-depth analysis to evaluate their morphology and porosity. The selection was based on the successful recovery of measurable and analyzable samples following the respective base treatments. Notably, the morphology of the recovered samples exhibited no discernible damage, as validated by the scanning electron microscope (SEM) images presented in Figure 8. Both the SEM images and the bulk crystallinity, as confirmed via the PXRD analysis depicted in Figure 7, indicated an overall influence on the entire MOF particles rather than the partial dissolution of specific MOF components.

**Figure 9:**
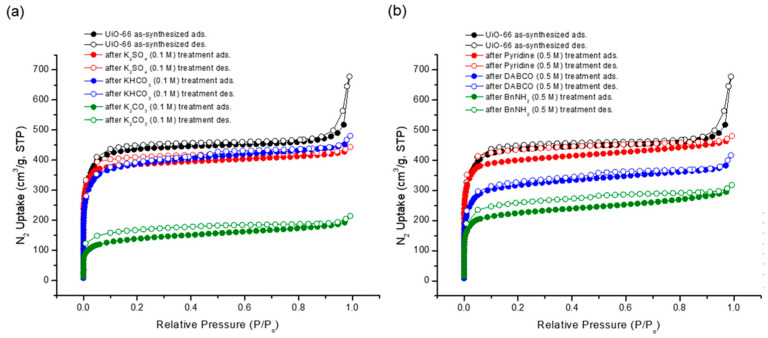
N_2_ adsorption (at 77 K) experiments of (**a**) recovered UiO-66 after inorganic base treatments and (**b**) recovered UiO-66 after organic base treatments.

Although the morphology of the MOF particles remained intact, the porosity experienced alterations due to the basic treatment. Gas adsorption experiments unveiled reductions in the N_2_ uptake following base treatments, with stronger bases demonstrating a more pronounced impact on porosity than their weaker counterparts (Figure 9). Notably, the N_2_ uptake underwent an approximate 50% decrease under the BnNH_2_ treatment among the organic bases. A subsequent Brunauer–Emmett–Teller (BET) surface area analysis was performed. While the pristine UiO-66 MOF boasted a BET surface area of 1777 m^2^/g, the recovered sample generally displayed a diminished surface area. Specifically, K_2_CO_3_ yielded the lowest surface area (524 m^2^/g), and weak bases like KHCO_3_, K_2_SO_4_, and pyridine had a minimal impact on the surface area (see Table 3).

Finally, inductively coupled plasma–optical emission spectrometry (ICP-OES) analysis was conducted to examine the metal-to-ligand ratio of the MOF following the base treatment. Given that the general formula of UiO-66 MOF is Zr_6_O_4_(OH)_4_(BDC-ligand)_6_, the dissolution of the ligand in a basic solution could lead to an increase in the metal ratio [72,73,74]. While UiO-66 exhibits a theoretical zirconium content of 32.9 wt%, the pristine UiO-66 (as-synthesized form) contains 34.0 wt% due to structural defects. Following the base treatment, strong bases, such as K_2_CO_3_ (inorganic) and BnNH_2_ (organic), exhibited significant increases in the Zr ratio according to ICP-OES analysis (refer to Table 4).

### 2.4. Comprehensive Concentration Limits and pK_b_ Values for UiO-66 MOF

**Figure 10:**
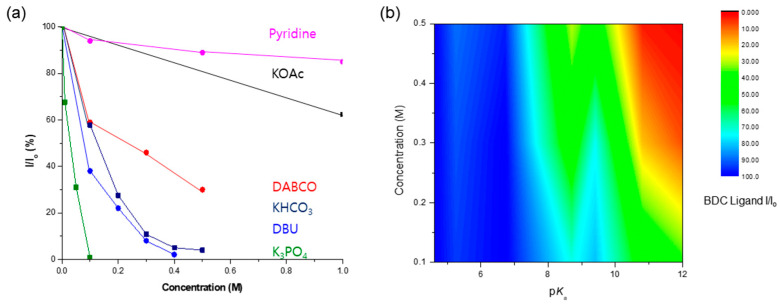
(**a**) Concentration limits of UiO-66 MOFs against the basic solution. (**b**) The correlation of p*K*_a_ and concentration for the conservation of the MOF structure.

A comprehensive investigation demonstrated that KOAc and pyridine were the bases with the highest concentration limits for Zr-based UiO-66 MOFs. As an organic base, pyridine demonstrated remarkable ligand preservation, with more than 80% remaining intact within the solid-state frameworks. Similarly, the KOAc treatment retained over 60% of the ligands, as shown in Figure 10. During the DABCO base test, approximately 70% of the BDC ligands were dissolved from the solid framework into solution. Despite ligand dissolution, the PXRD patterns were adequately retained, and the morphology of UiO-66 remained intact.

The relationship between the strength of the base (expressed by the p*K*_b_ value) and concentration was directly linked to the dissolution of the MOF frameworks. High basicity (i.e., low p*K*_b_ and high p*K*_a_) proved critical for MOF destruction owing to a strong nucleophilic attack, while high concentrations also played a crucial role in MOF dissolution. Figure 10b illustrates the correlation between p*K*_a_ and the concentration of the basic solution. The red color signifies the MOF framework dissolution, whereas the blue portion denotes the conserved structure of the MOFs. This visual representation underscores the interplay between the base strength and concentration in determining the fate of MOF stability under basic conditions.

## 3. Conclusions

Zr-based MOFs have gained widespread attention and applications owing to their excellent chemical and physical stability, ease of accessibility, and tunability. However, the practical deployment of these MOFs in industrial and daily-life scenarios is constrained by their inherent susceptibility to basic conditions. This limitation is indeed critical in a plethora of organic syntheses that use basic additives.

This study systematically probed the stability of Zr-based UiO-66 MOFs in various basic solutions, in which inorganic and organic bases commonly found in chemistry laboratories were investigated. The findings revealed that highly basic solutions (p*K*_b_ < 3.3) led to the complete destruction of UiO-66 MOFs within 1 h at a 0.1 M concentration. Subsequent examinations focused on bases that have a higher p*K*_b_ than 3.3. Among these experiments, UiO-66 MOFs exhibited overall stability in both the KOAc and pyridine solutions, while PXRD and NMR analyses detected some framework destruction and ligand dissolution during the KHCO_3_ and DABCO solution treatments. Notably, K_3_PO_4_ and DBU exhibited low concentration limits for the degradation of the UiO-66 MOF.

In conclusion, when designing experiments involving Zr-MOFs under basic conditions, considering the concentration and p*K*_b_ values of the chosen base is essential. In this context, we believe that Figure 10 can serve as a key guide to practitioners in selecting bases for UiO-66 MOFs under aqueous conditions. Notably, MOF stability may also be influenced by metal salts, ligand functionalization, solvents, etc. Therefore, additional systematic consideration, both from the literature and empirical perspectives, is imperative for advancing the practical applications of MOFs.

## Figures and Tables

**Table 1:** p*K*_b_ value of tested inorganic bases toward UiO-66 MOF.

Base	KOH	K_3_PO_4_	K_2_CO_3_	KHCO_3_	KOAc	K_2_SO_4_
p*K*_b_	−1.7	1.6	4.1	7.2	9.2	12

**Table 2:** p*K*_b_ values of the tested organic bases toward the UiO-66 MOF.

Base	DBU	Et_3_N	BnNH_2_	DABCO	Pyridine
p*K*_b_	0.5	3.3	4.6	5.2	8.8

**Table 3:** BET surface area of recovered UiO-66 after base treatments.

Base	- (Pristine)	K_2_CO_3_ (0.1 M)	KHCO_3_ (0.1 M)	K_2_SO_4_ (0.1 M)	BnNH_2_ (0.5 M)	DABCO (0.5 M)	Pyridine (0.5 M)
BET surface area (m^2^/g)	1777	524	1540	1566	881	1060	1622

**Table 4:** The ICP-OES of recovered UiO-66 after base treatments.

Base	- (Pristine)	K_2_CO_3_ (0.1 M)	KHCO_3_ (0.1 M)	K_2_SO_4_ (0.1 M)	BnNH_2_ (0.5 M)	DABCO (0.5 M)	Pyridine (0.5 M)
Zr wt%	34.0	45.2	25.7	30.0	49.4	31.0	35.6

## Data Availability

Data will be available upon request.

## Supplementary Materials

The following supporting information can be downloaded at: https://www.mdpi.com/article/10.3390/nano14010110/s1, General methods, preparation of UiO-66, procedure of base treatment, PXRD measurement, acid digestion of MOFs for NMR measurement, N_2_ isotherm for UiO-66, and appendix-PXRD and ^1^H NMR data of UiO-66. Ref. [63] is cited in the supplementary materials.
