# Supplementary material for: Stability of Zr-Based UiO-66 Metal–Organic Frameworks in Basic Solutions
Source: Nanomaterials (Basel). 2024 Jan 2;14(1):110. doi: 10.3390/nano14010110 (PMC10780619; doi:10.3390/nano14010110)

Supporting Information

Stability of Zr-Based UiO-66 Metal–Organic Frameworks in Basic Solutions

Jun Yeong Kim [1,†], Jiwon Kang [1,†], Seungheon Cha [1], Haein Kim [1], Dopil Kim [1], Houng Kang [2], Isaac Choi [1,*] and Min Kim [1,*]

[1] Department of Chemistry, Chungbuk National University, Cheongju 28644, Republic of Korea; junyeongkim1111@gmail.com (J.Y.K.); kangjiwon1001@gmail.com (J.K.); chaseungheon045@gmail.com (S.C.); haeinkim9951@gmail.com (H.K.); ehvlfdl0218@gmail.com (D.K.)

[2] Department of Chemistry Education, Chungbuk National University, Cheongju 28644, Republic of Korea; hkang@chungbuk.ac.kr

* Correspondence: isaac.choi@chungbuk.ac.kr (I.C.); minkim@chungbuk.ac.kr (M.K.)

† These authors contributed equally to this work.

S1. General Methods

All reagents and solutions used in this study were obtained from Sigma-Aldrich (St. Louis, MO, USA), TCI (Olive Branch, MS, USA), Alfa-Aesar (Haverhill, MA, USA), and Acros companies (Vadodara, India). All reagents and solvents were directly used without further purification. ^{1}H Nuclear Magnetic Resonance (NMR) spectra was acquired using Magritek Spinsolve 80 MHz Ultra in Chungbuk National University. Powder X-ray diffraction (PXRD) was measured using Rigaku Miniflex 600 in Chungbuk National University. Scanning electron microscope images were taking using ZEISS Ultra Plus Field Emission SEM in the center for research facilities at Chungbuk National University .

S2. Preparation of UiO-66

UiO-66 MOF was prepared by following Farha's method with modification [S1]. Terephthalic acid (BDC, 83 mg, 0.5 mmol), $ZrCl_4$ (110 mg, 0.47 mmol), formic acid (1.67 mL), DMF (14 mL) and H_2O (20 μL) were placed in a 20 mL vial, and sonicated until solids were fully dissolved. Then solution mixture was heated in a conventional oven at 120 °C for 24 h. After cooling to room temperature, the colorless, microcrystalline powder was isolated by centrifugation (3600 rpm). The isolated powder was washed 3 times with fresh DMF (3 x 10 mL), and then washed with MeOH (3 x 10 mL). In every washing step, the soaking interval (30 minutes) is necessary for full diffusion of solvent into the pore of MOFs. The solids were dried under vacuum to check stability.

S3. Procedure of Base Treatment

Fully dried UiO-66 sample (as synthesized, 30 mg) was placed in a 4 mL vial and soaked in 3 mL of base solution. After incubation time, MOF powder was isolated through centrifugation (3600 rpm) from basic solution. The obtained solid was washed 3 times with deionized water (3 x 1 mL) and methanol (3 x 1 mL). The remaining solvent was dried under vacuum.

S4. PXRD measurement

Approximately 10 mg of MOF samples were measured at room temperature on a Rigaku Miniflex 600 (40 kV & 15 mA for CuKa, λ = 1.5406 Å), with a scan speed of 10 sec/step, a step size of 0.02° in 2θ, and a 2θ range of 4.5-30.5° to check bulk crystallinity.

S5. Acid digestion of MOFs for NMR measurement

Approximately 10 mg of UiO-66 samples after PXRD analysis were dried under vacuum for overnight, and fully dried samples were digested in the solution mixture of 590 μL of DMSO-d_6 and 10 μL of HF (48% aqueous solution) with sonication until full dissolution (5-30 minutes).

S6. N_2 full isotherms for UiO-66

Approximately 40 mg of UiO-66 samples were evacuated under vacuum at room temperature. Then samples were

transferred to a pre-weighed sample tube, and degassed at 150 ºC on an ASAP2020 for a minimum of 24 h or until the outgas rate was < 5 μm Hg/min. The sample tube was re-weighted to obtain a consistent mass for the degassed MOF materials. Then N_2 isotherm measurements were performed at 77 K.

Reference for supporting information

[S1] Katz, M. J.; Brown, Z. J.; Colón, Y. J.; Siu, P. W.; Scheidt, K. A.; Snurr, R. Q.; Hupp, J. T.; Farha, O. K. A facile synthesis of UiO-66, UiO-67 and their derivatives. *Chemical Communications* **2013**, 49 (82), 9449-9451, 10.1039/C3CC46105J. DOI: 10.1039/C3CC46105J.

APPENDIX

PXRD and ^{1}H NMR data of UiO-66 treated with other bases

Treatment with NaOH

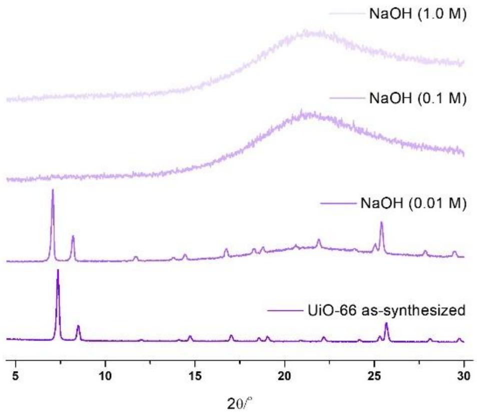

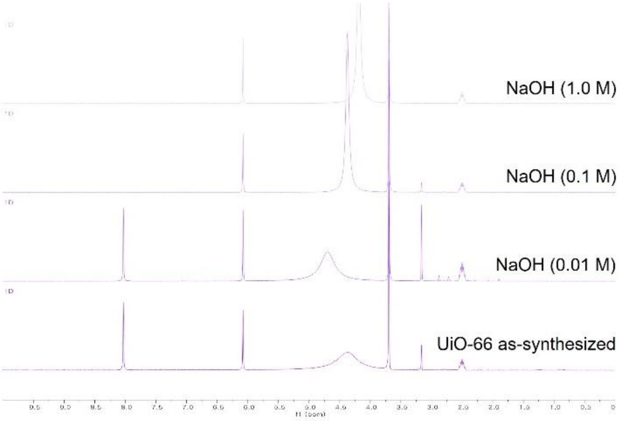

Treatment with Et_3N

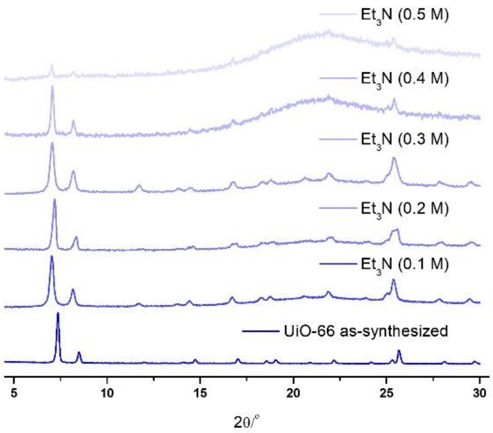

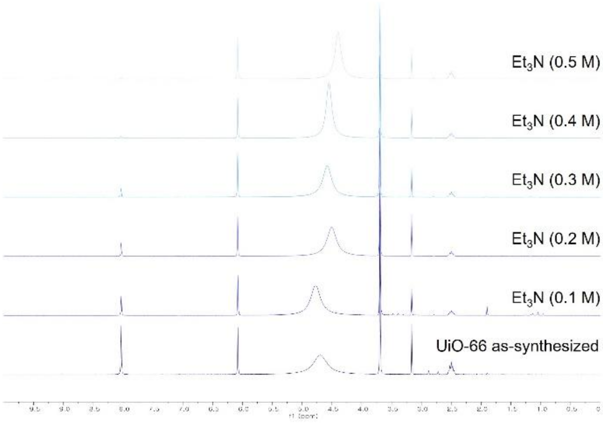

Treatment with aniline

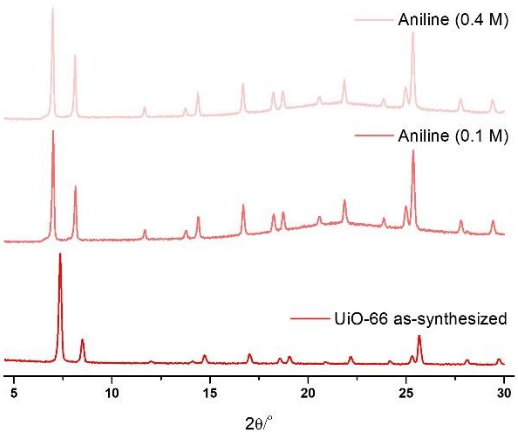

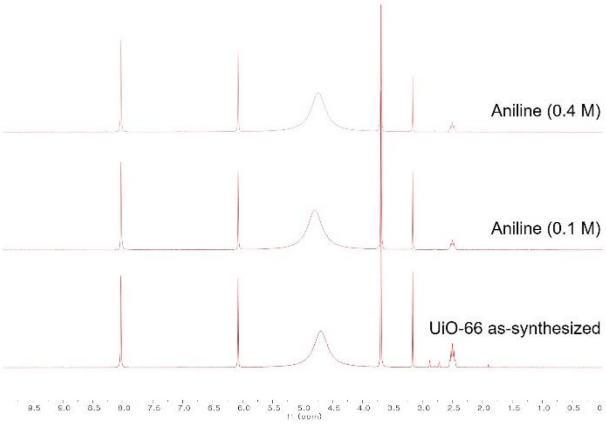

Treatment with 2,6-lutidine

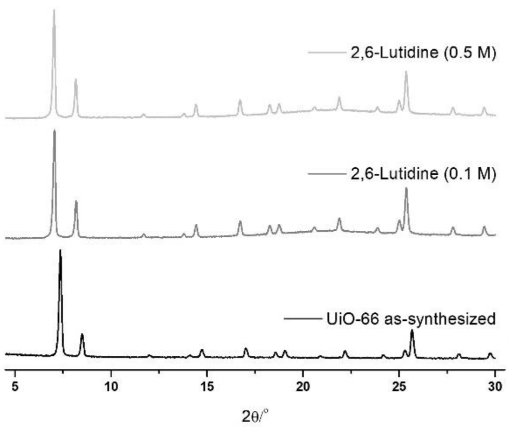

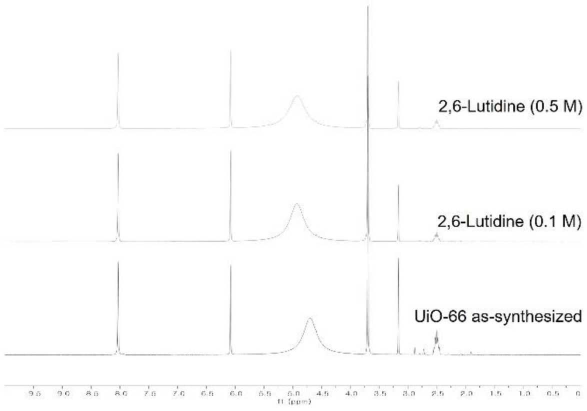

Treatment with benzylamine

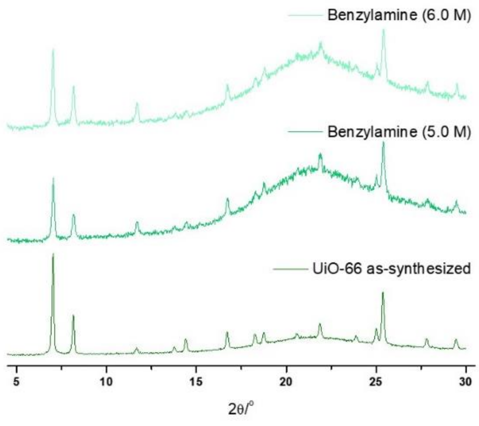

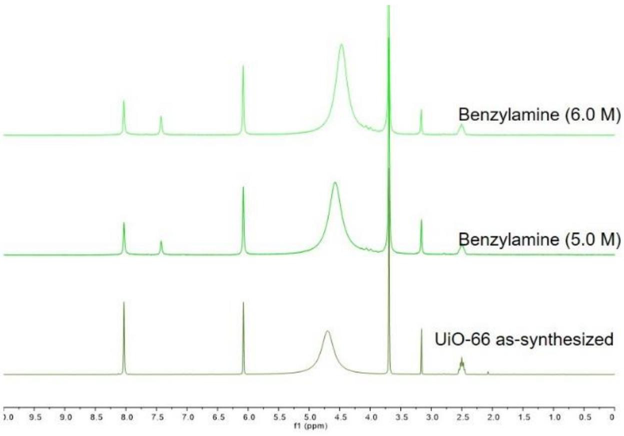

Peak integration with internal standard for treatment with NaOH 0.01 M

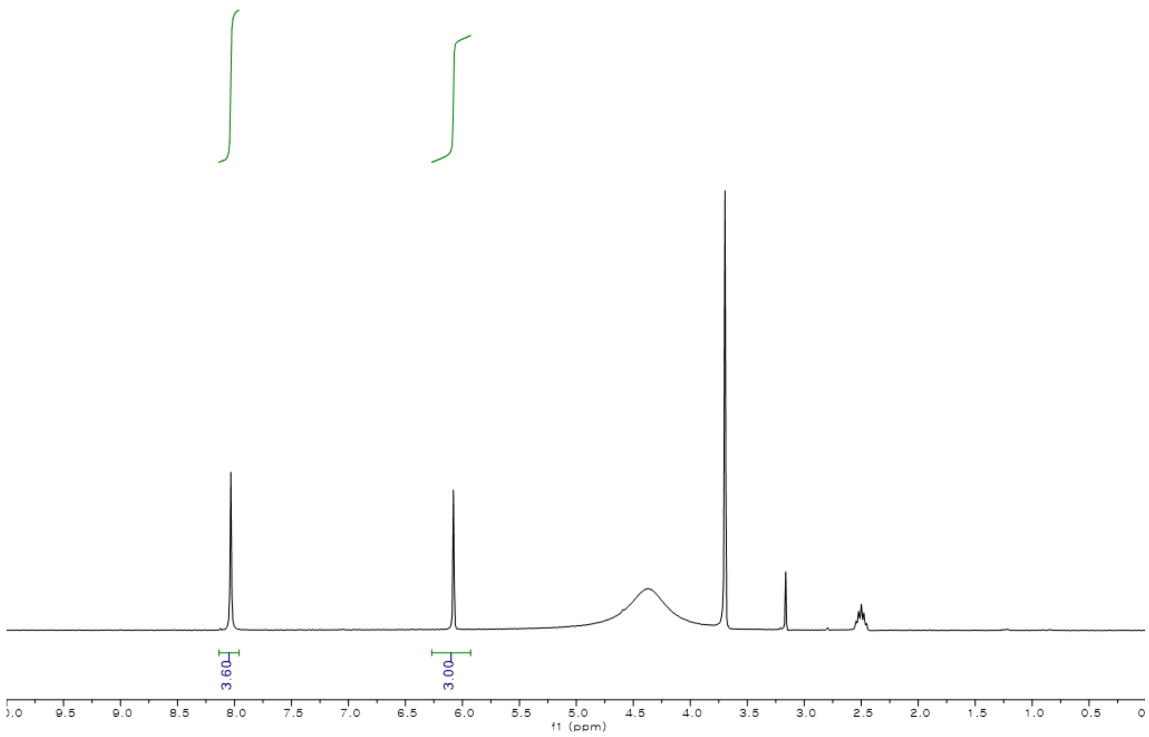

Peak integration with internal standard for treatment with NaOH 0.1 M

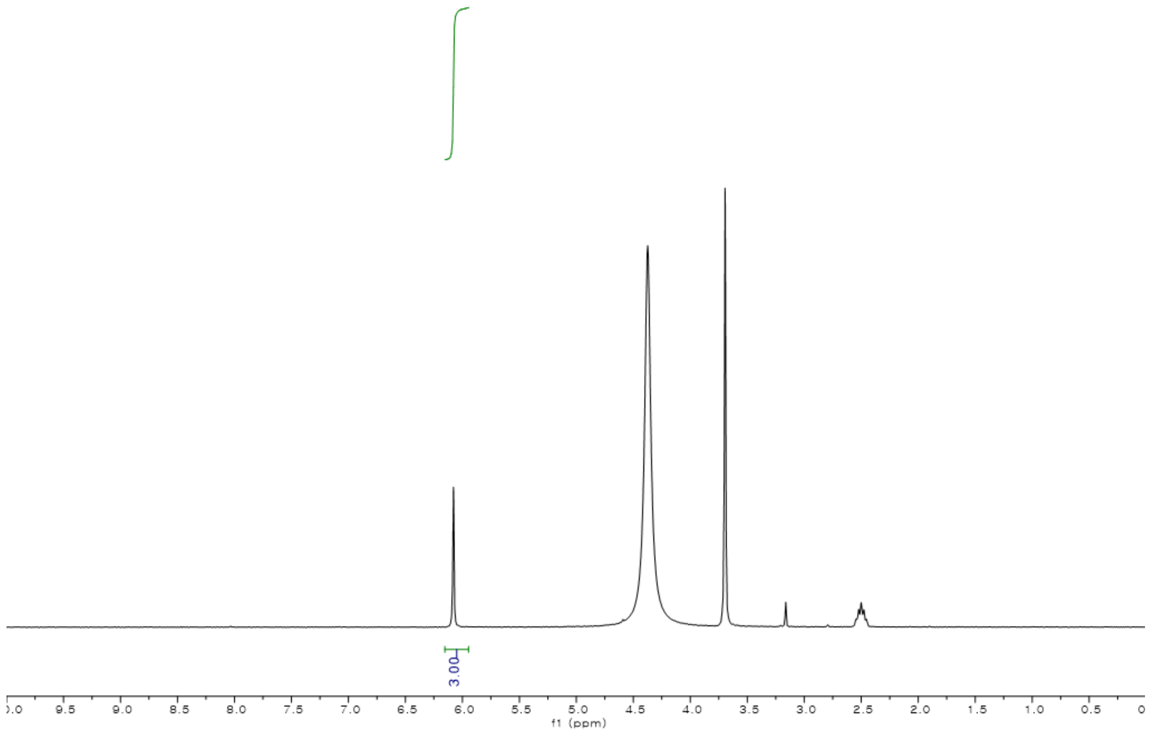

Peak integration with internal standard for treatment with NaOH 1.0 M

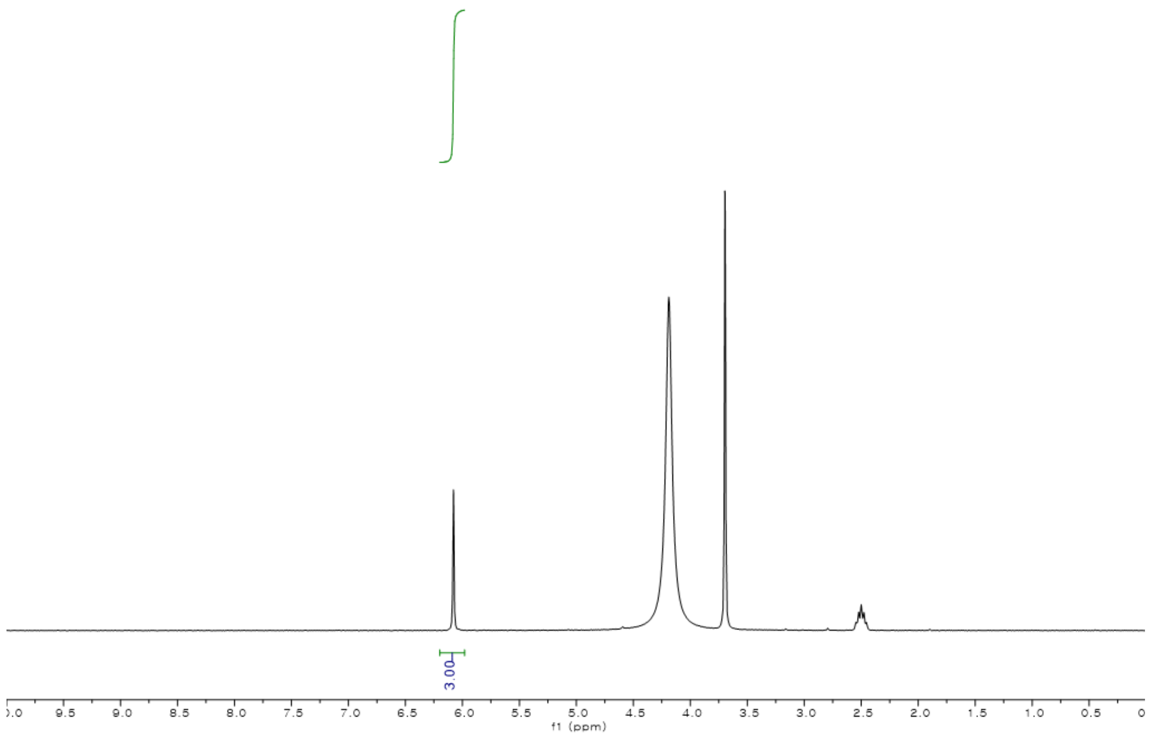

Peak integration with internal standard for treatment with KOAc 1.0 M

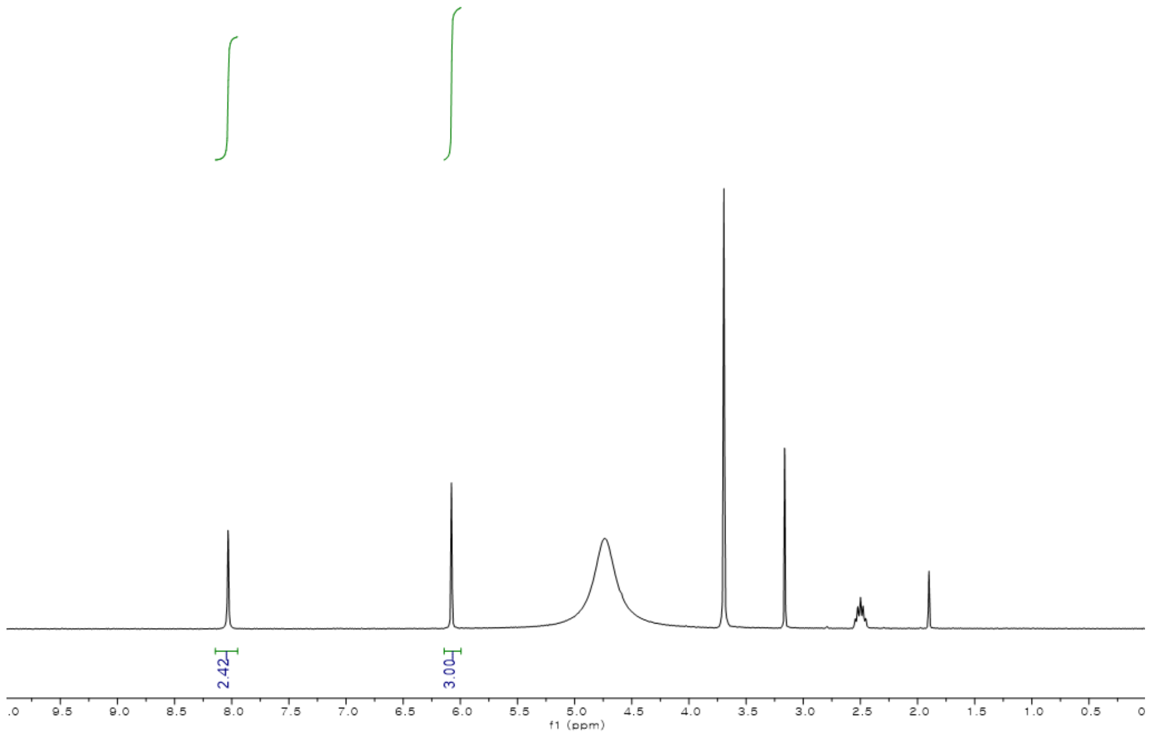

Peak integration with internal standard for treatment with KOAc 2.0 M

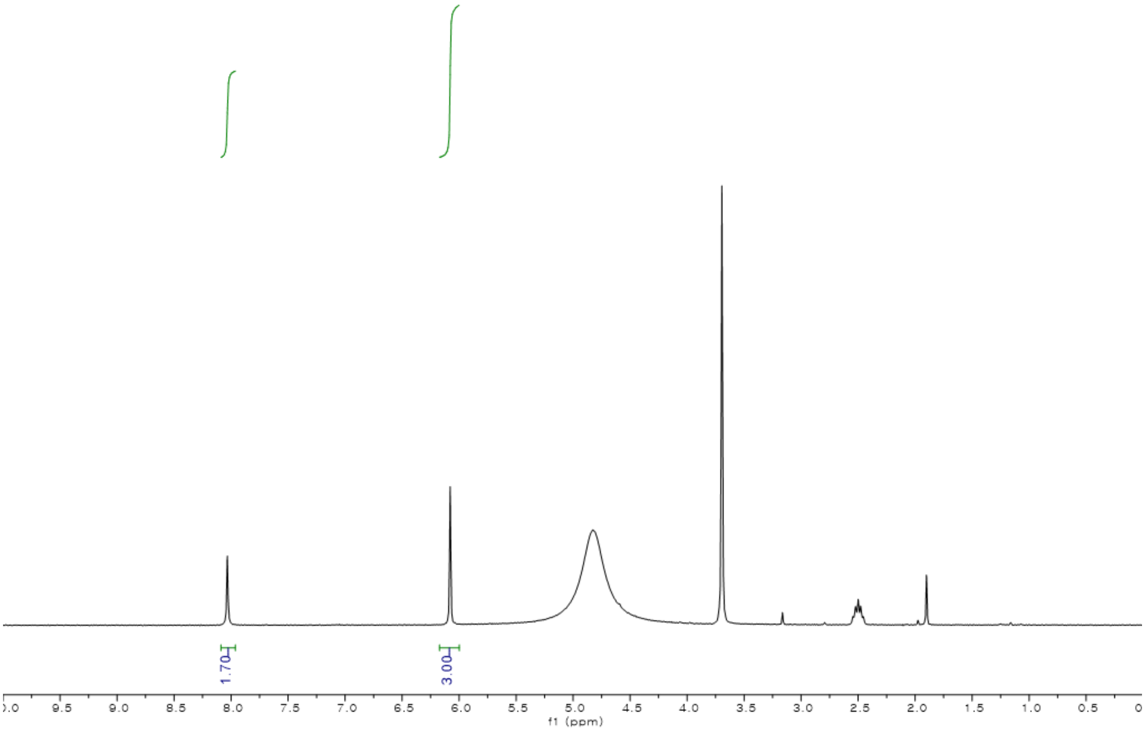

Peak integration with internal standard for treatment with KOAc 3.0 M

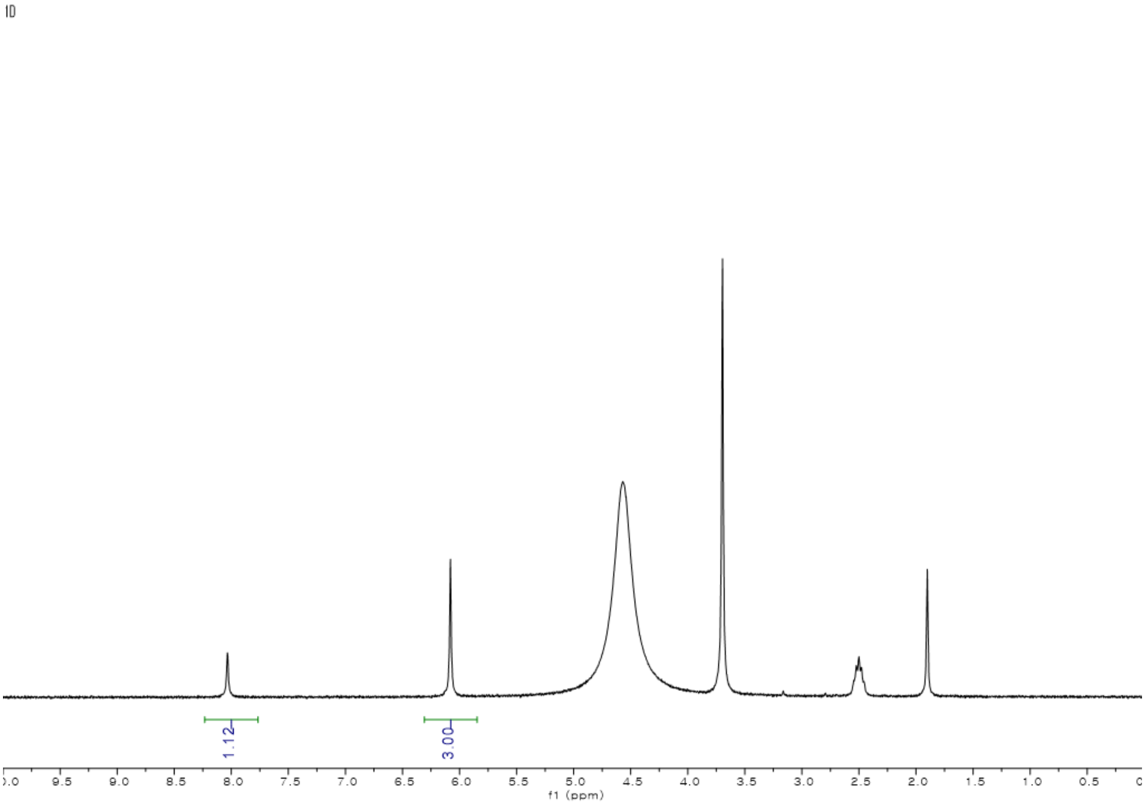

Peak integration with internal standard for treatment with $KHCO_3$ 0.1 M

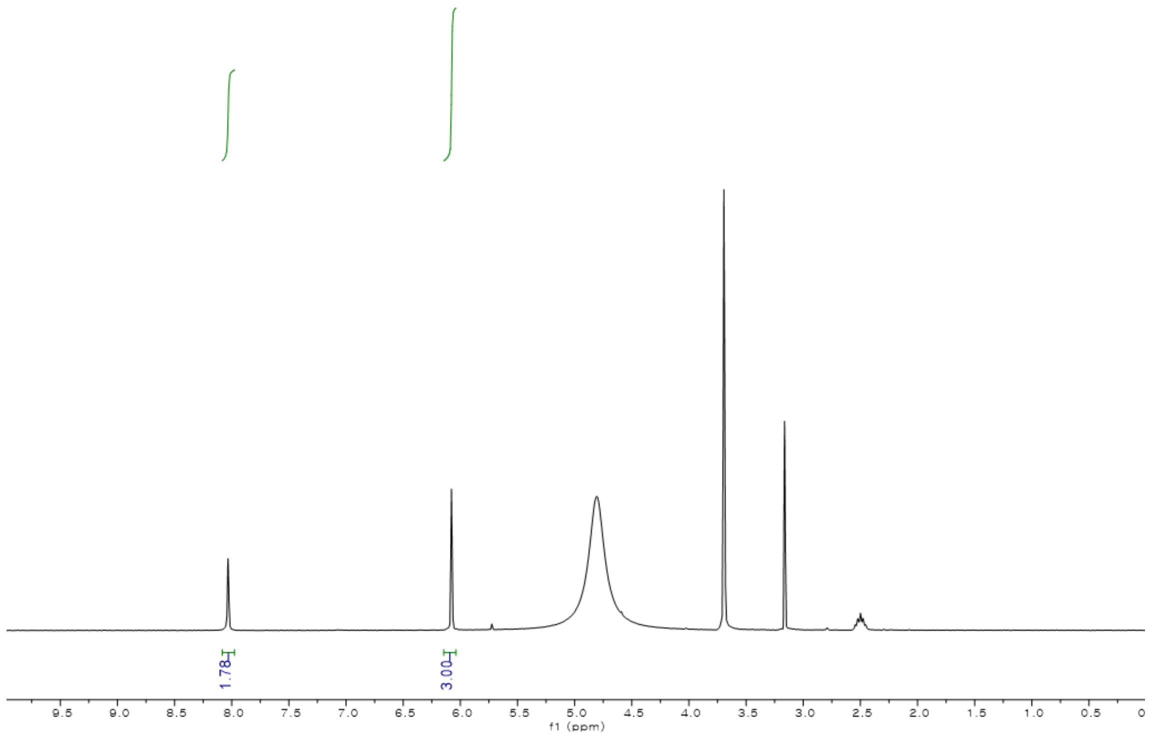

Peak integration with internal standard for treatment with $KHCO_3$ 0.2 M

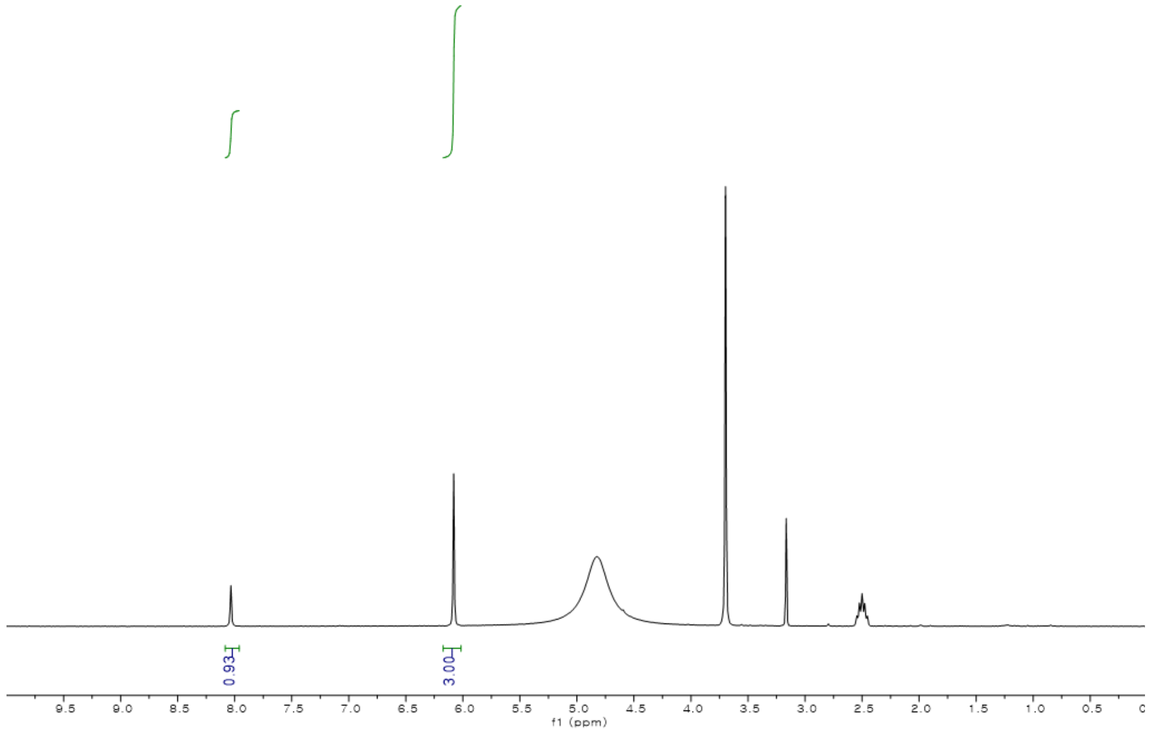

Peak integration with internal standard for treatment with $KHCO_3$ 0.3 M

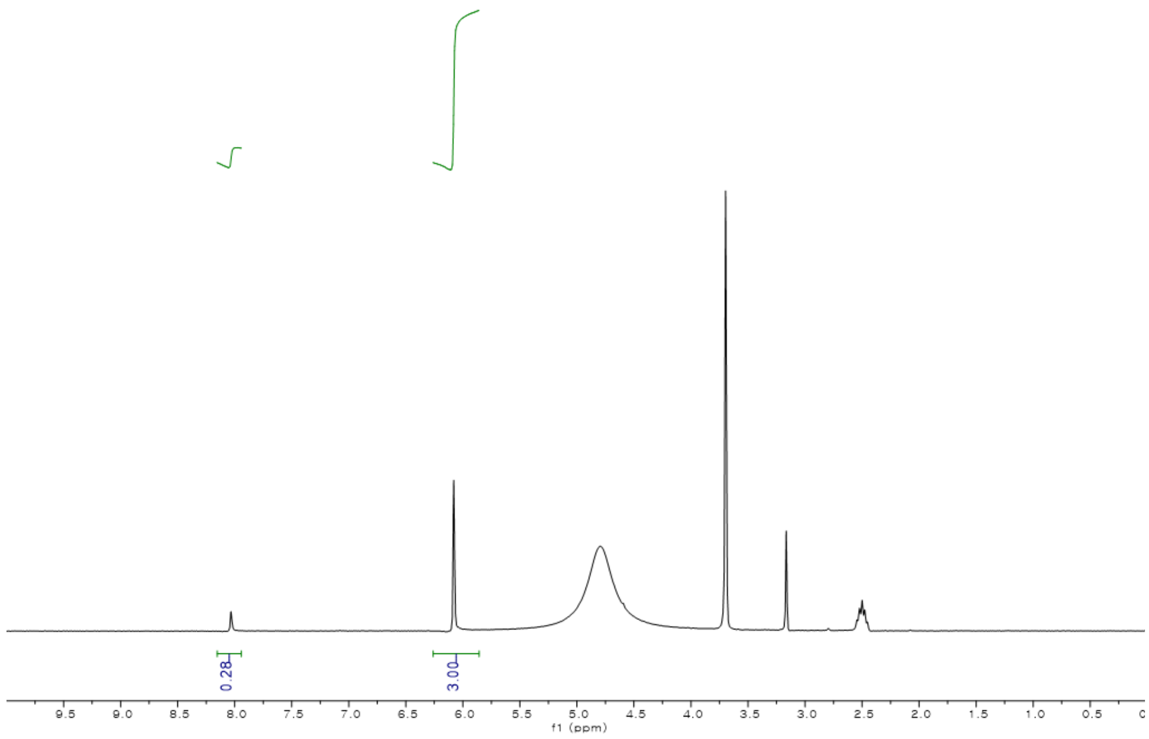

Peak integration with internal standard for treatment with $KHCO_3$ 0.4 M

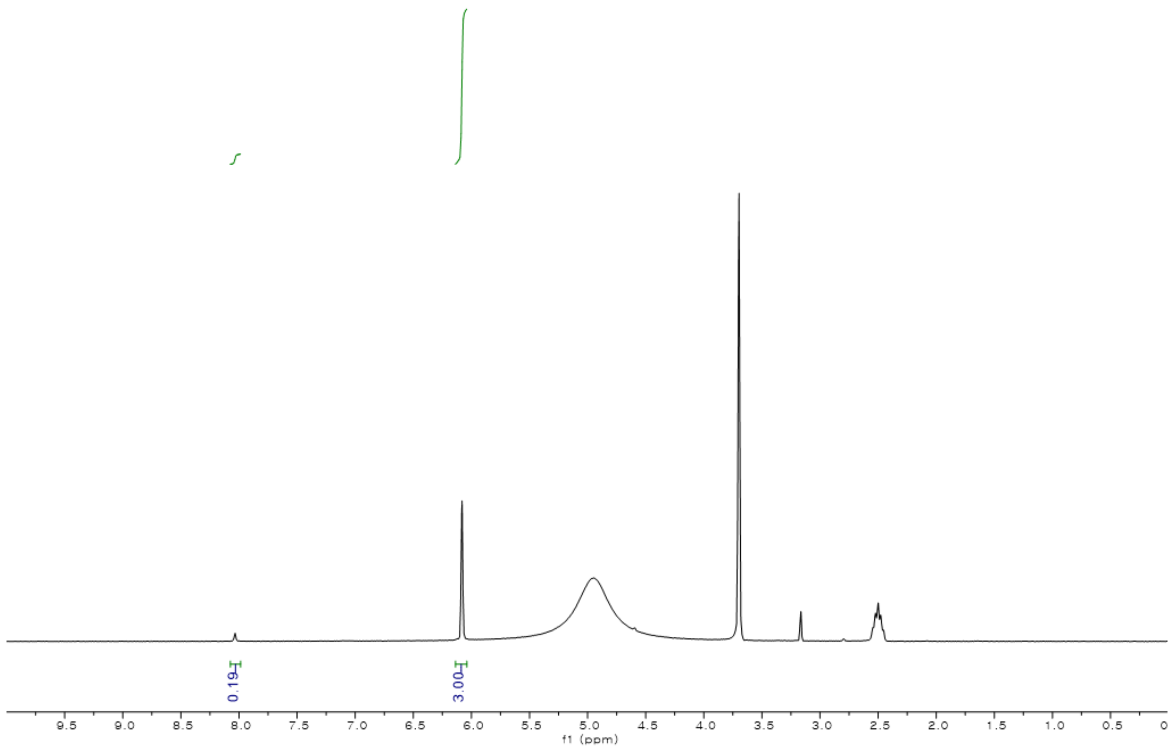

Peak integration with internal standard for treatment with K_3PO_4 0.01 M

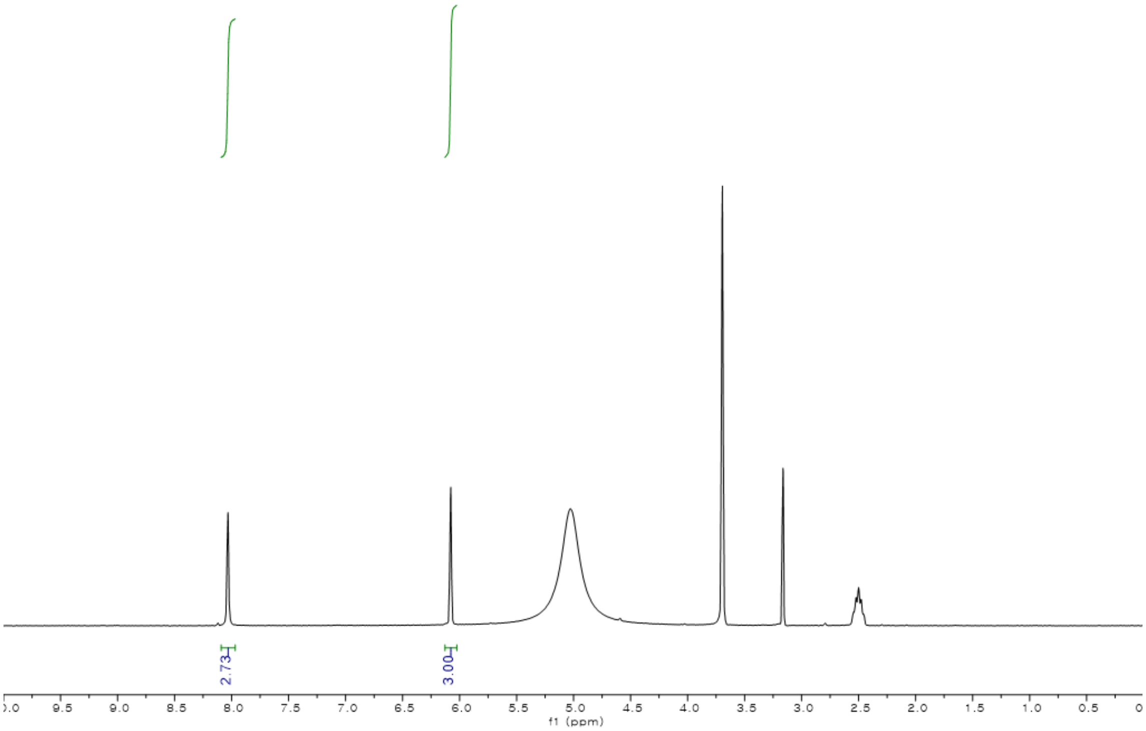

Peak integration with internal standard for treatment with K_3PO_4 0.05 M

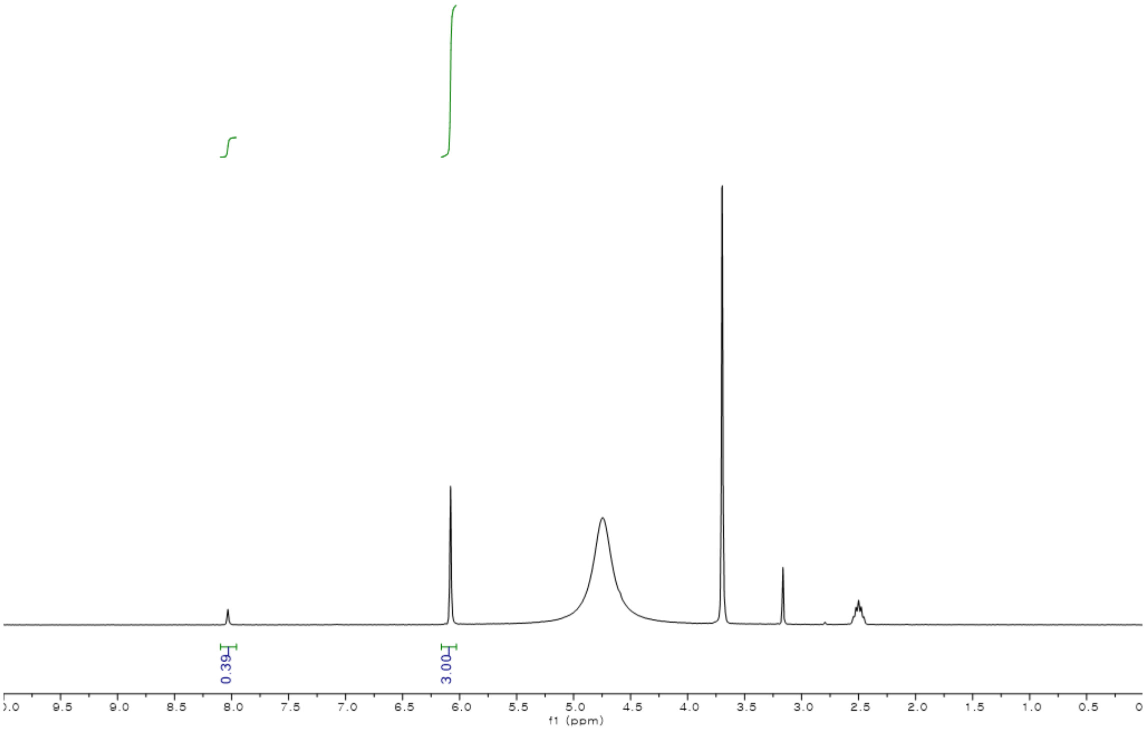

Peak integration with internal standard for treatment with K_3PO_4 0.1 M

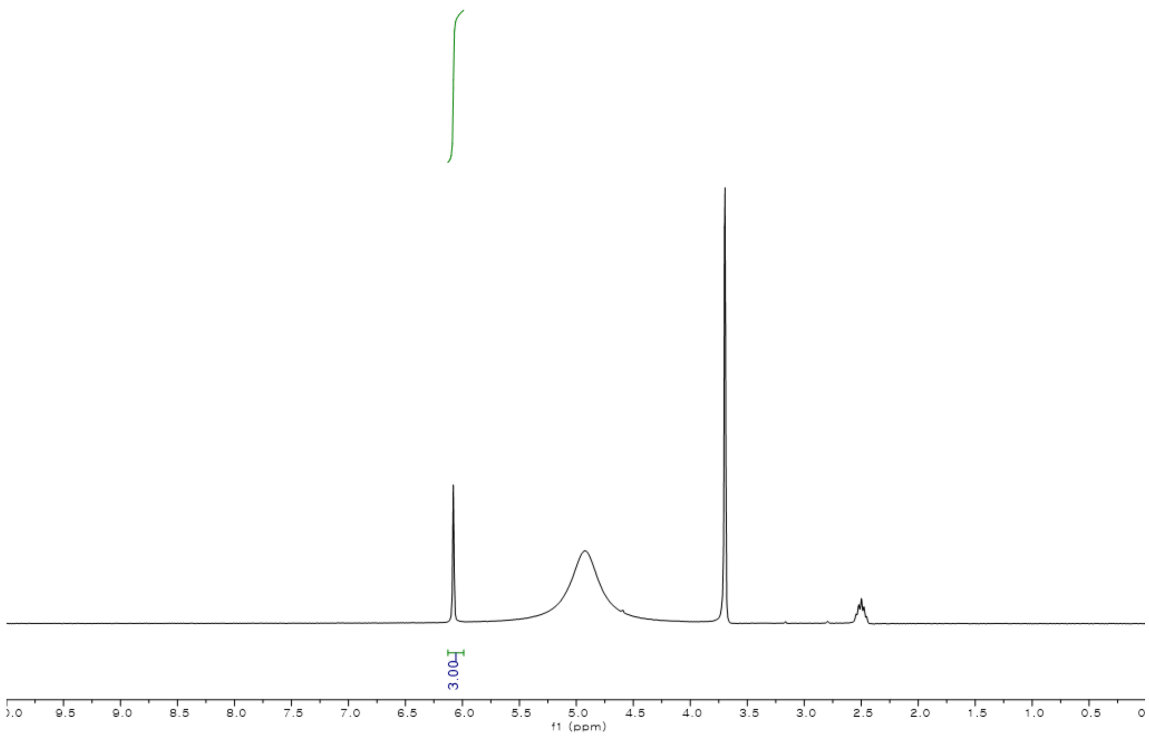

Peak integration with internal standard for treatment with DBU 0.1 M

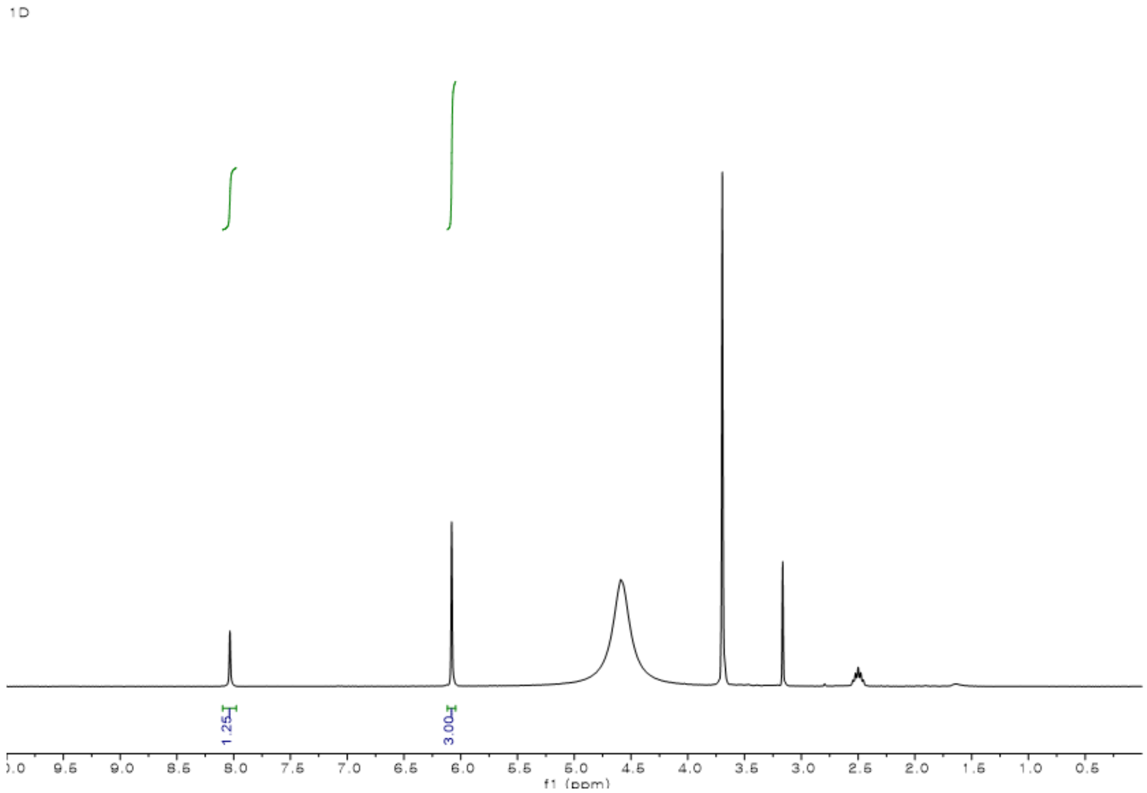

Peak integration with internal standard for treatment with DBU 0.2 M

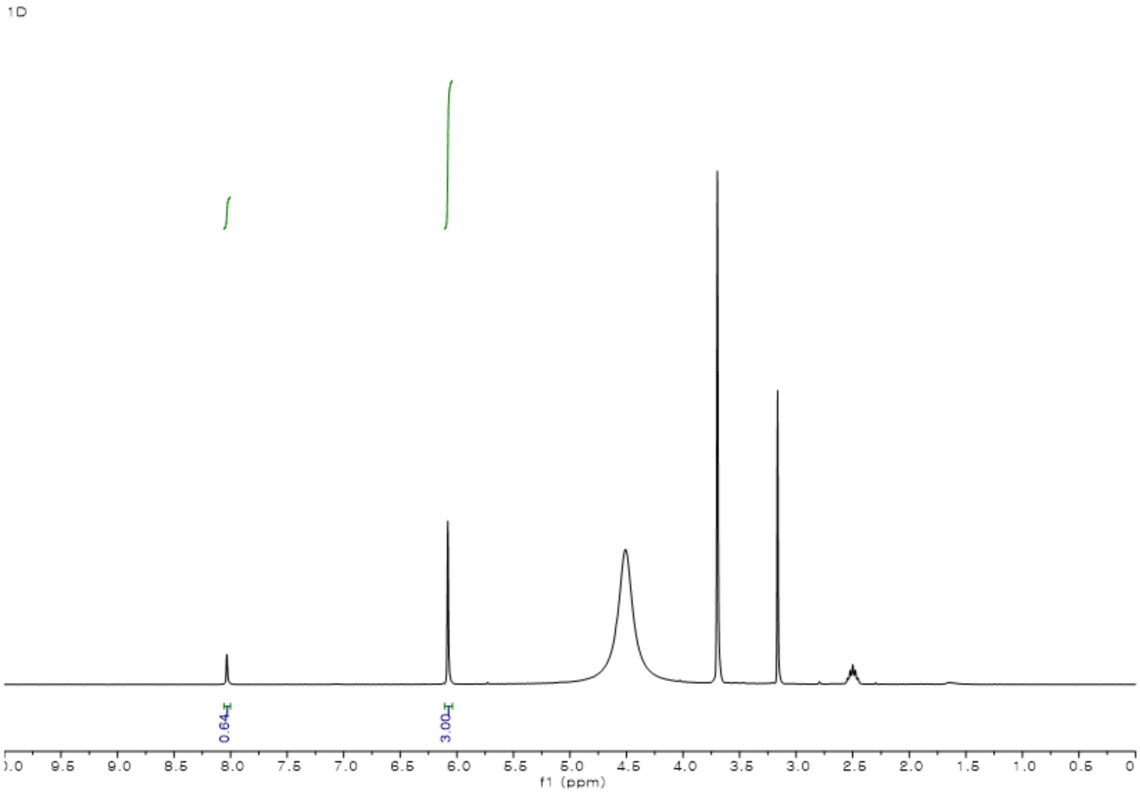

Peak integration with internal standard for treatment with DBU 0.3 M

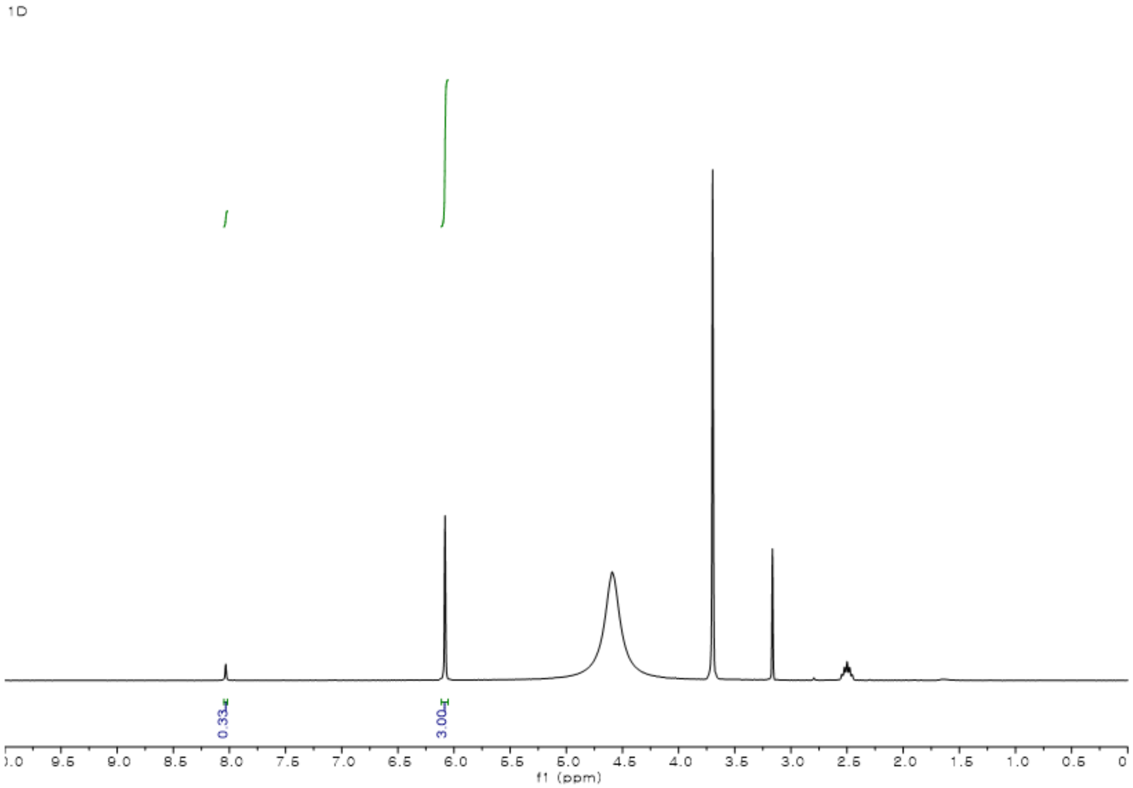

Peak integration with internal standard for treatment with DBU 0.4 M

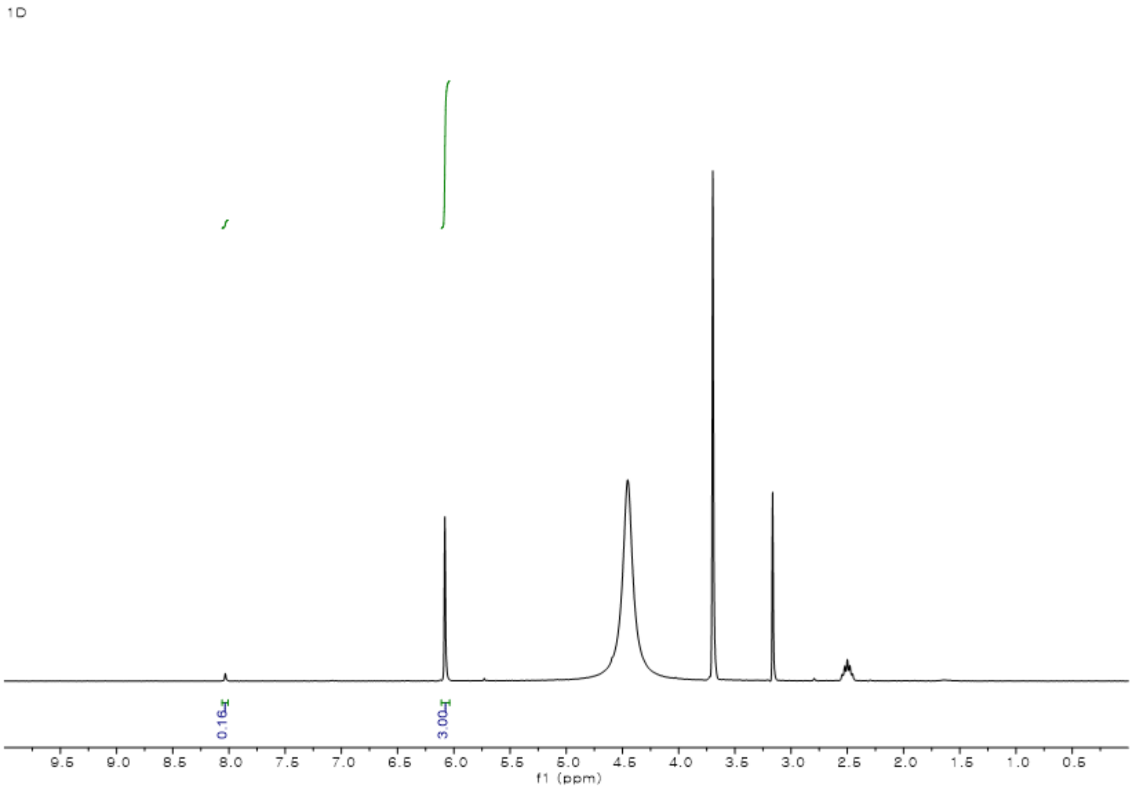

Peak integration with internal standard for treatment with DABCO 0.1 M

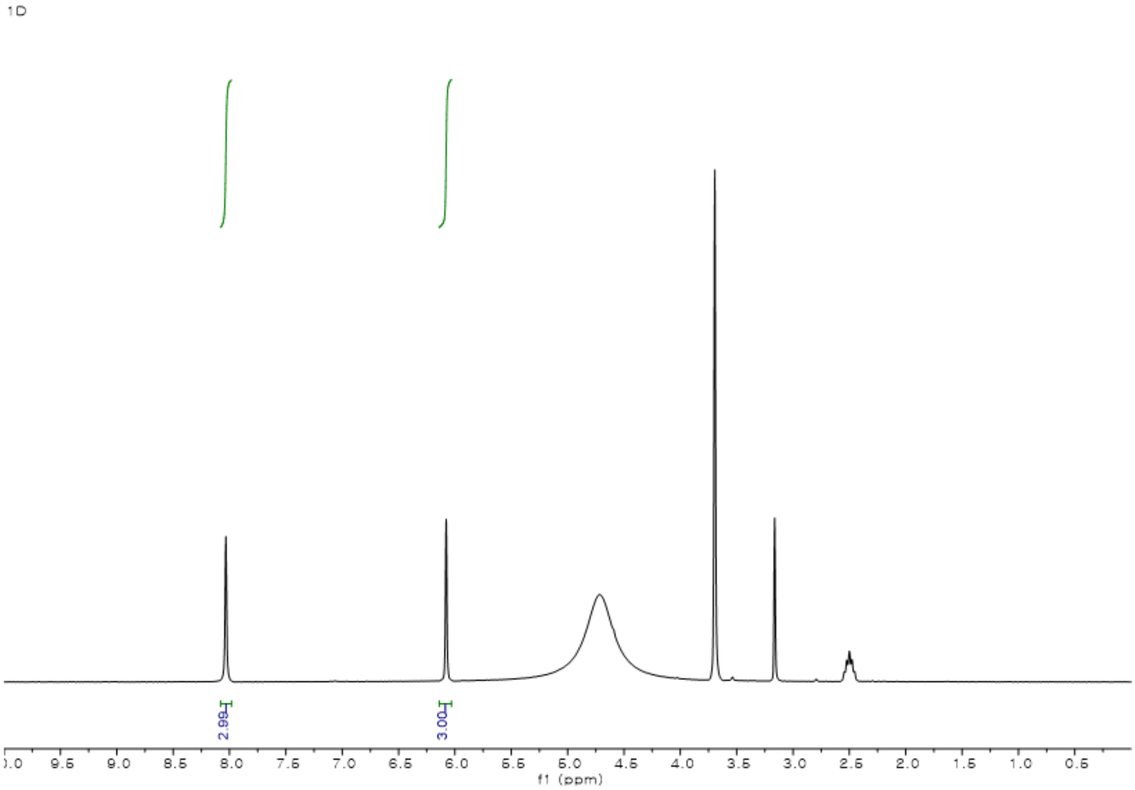

Peak integration with internal standard for treatment with DABCO 0.3 M

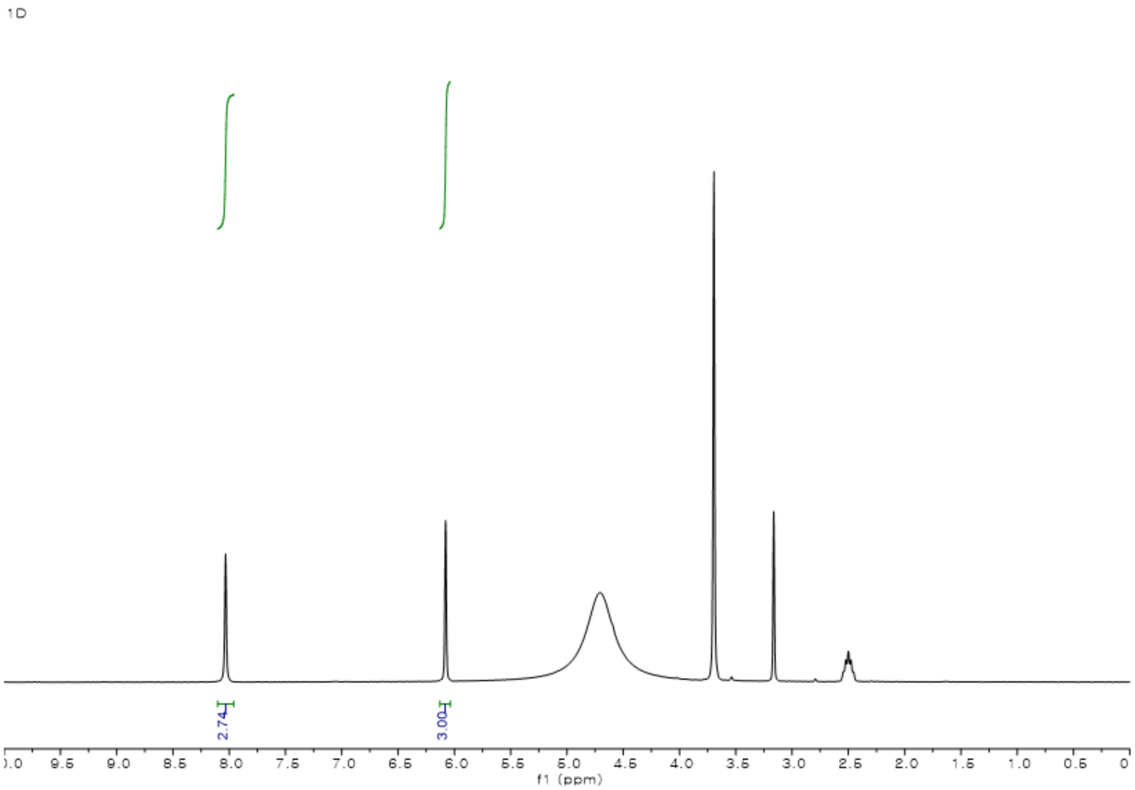

Peak integration with internal standard for treatment with DABCO 0.5 M

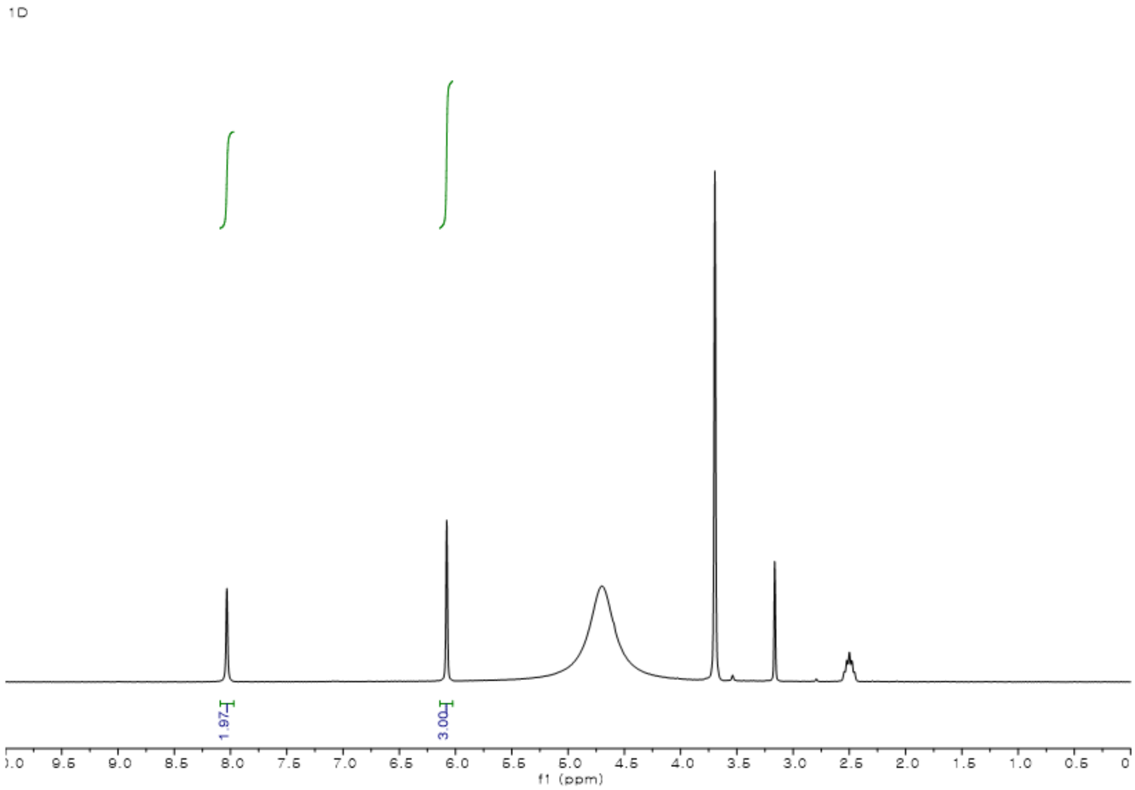

Peak integration with internal standard for treatment with pyridine 0.1 M

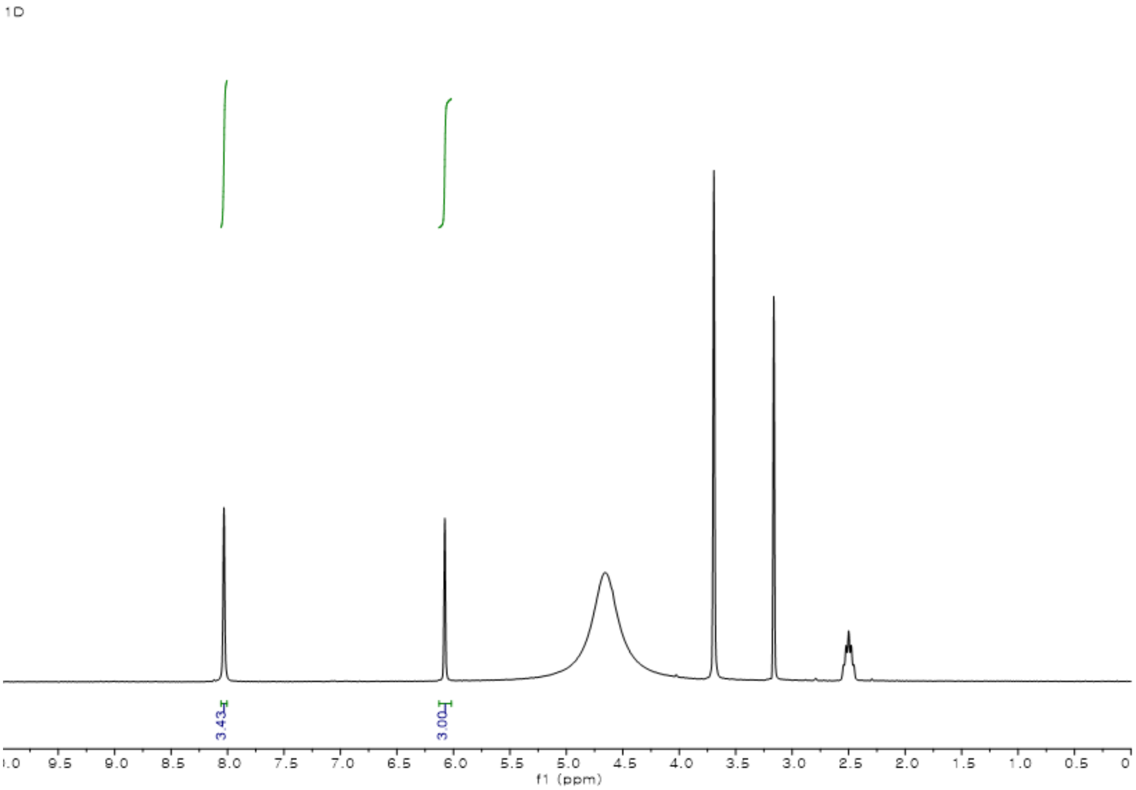

Peak integration with internal standard for treatment with pyridine 0.5 M

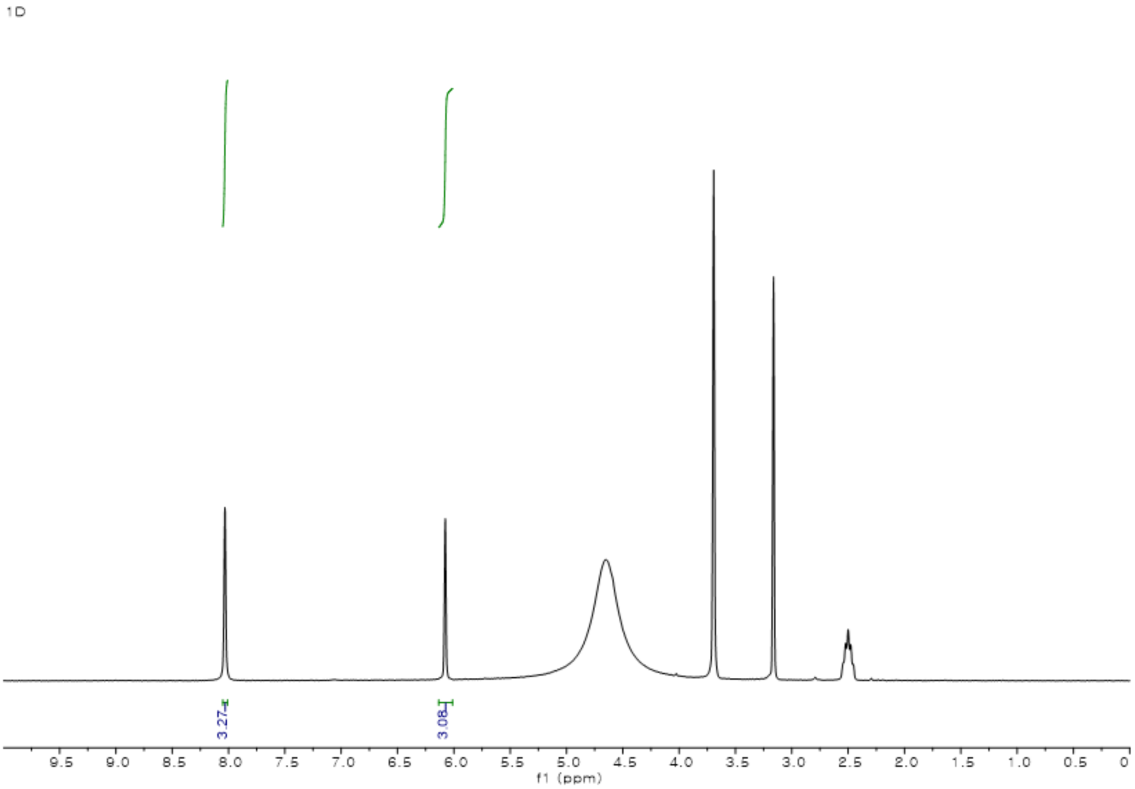

Peak integration with internal standard for treatment with pyridine 1.0 M

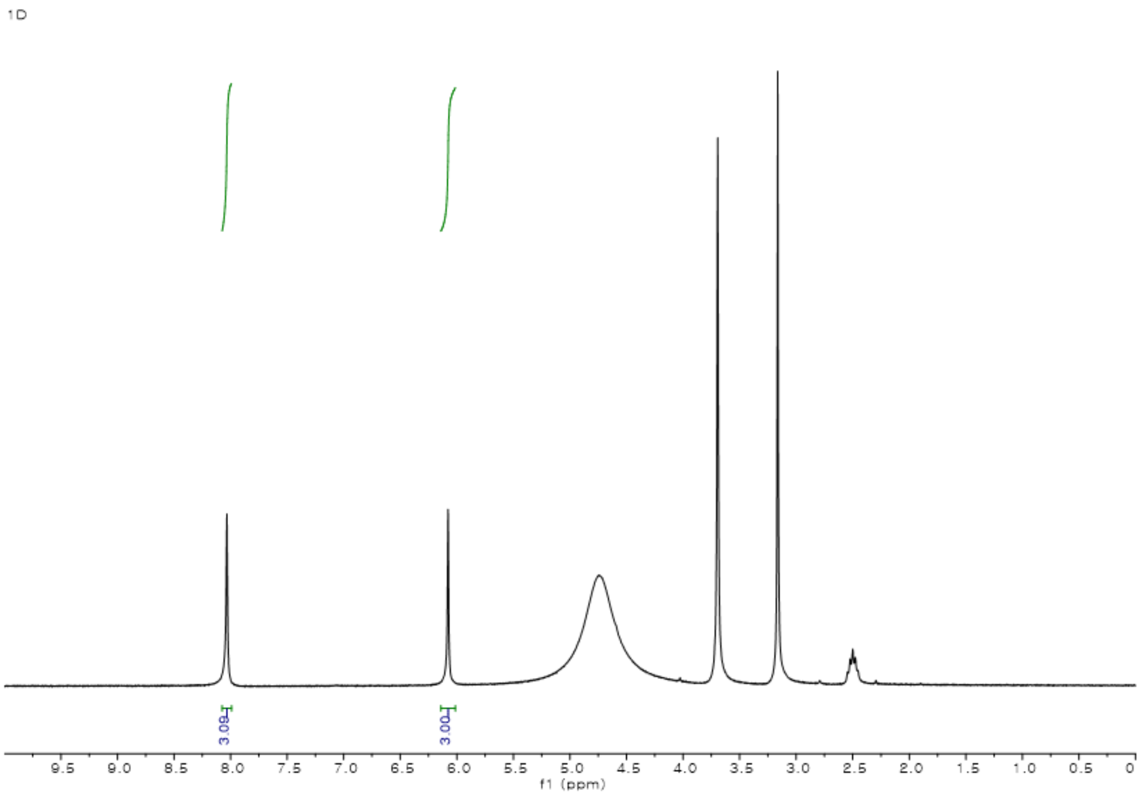

Peak integration with internal standard for treatment with Et_3N 0.1 M

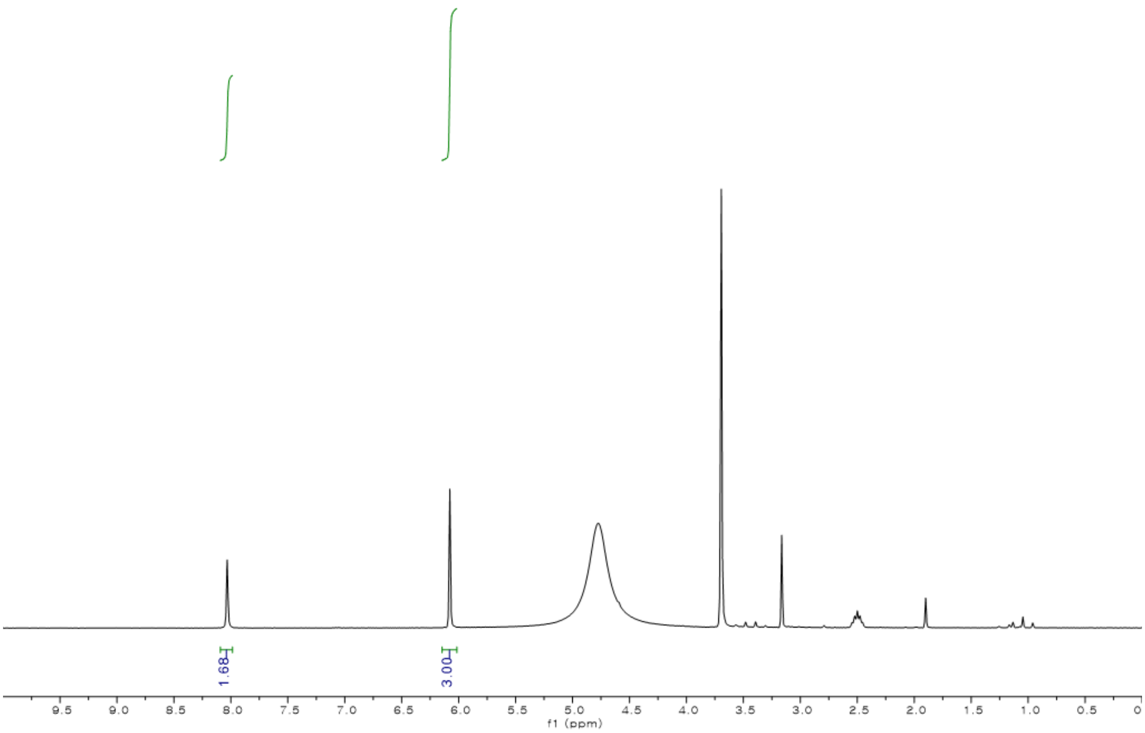

Peak integration with internal standard for treatment with Et_3N 0.2 M

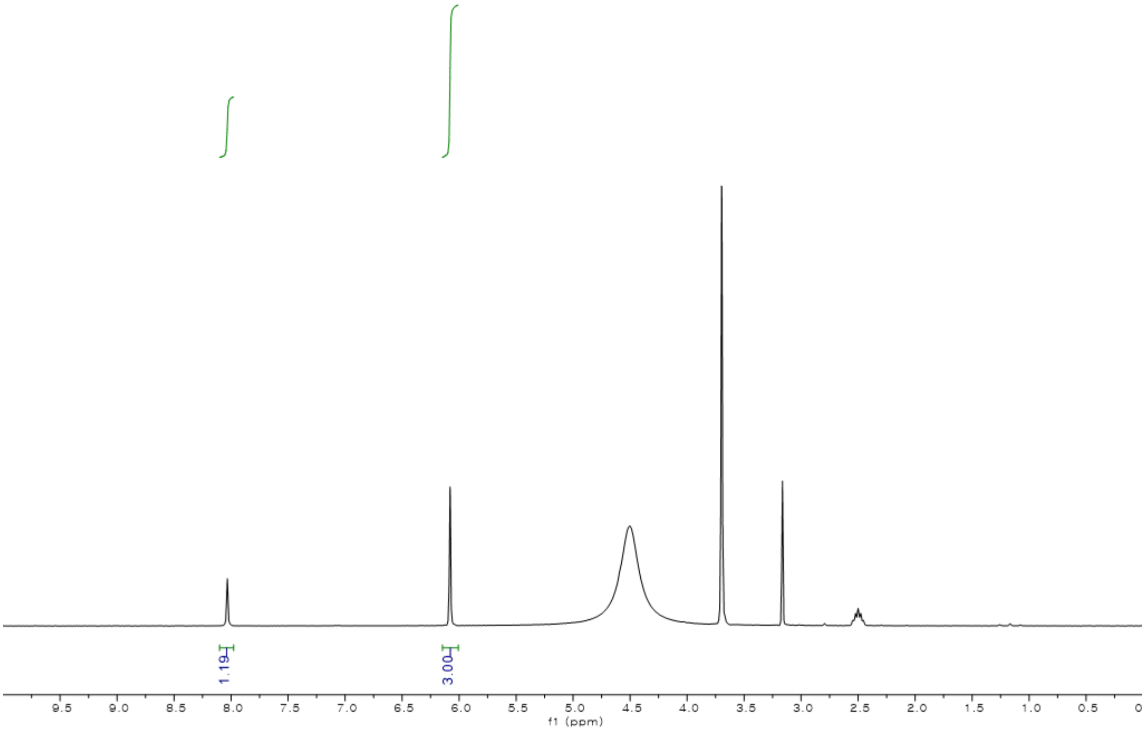

Peak integration with internal standard for treatment with Et_3N 0.3 M

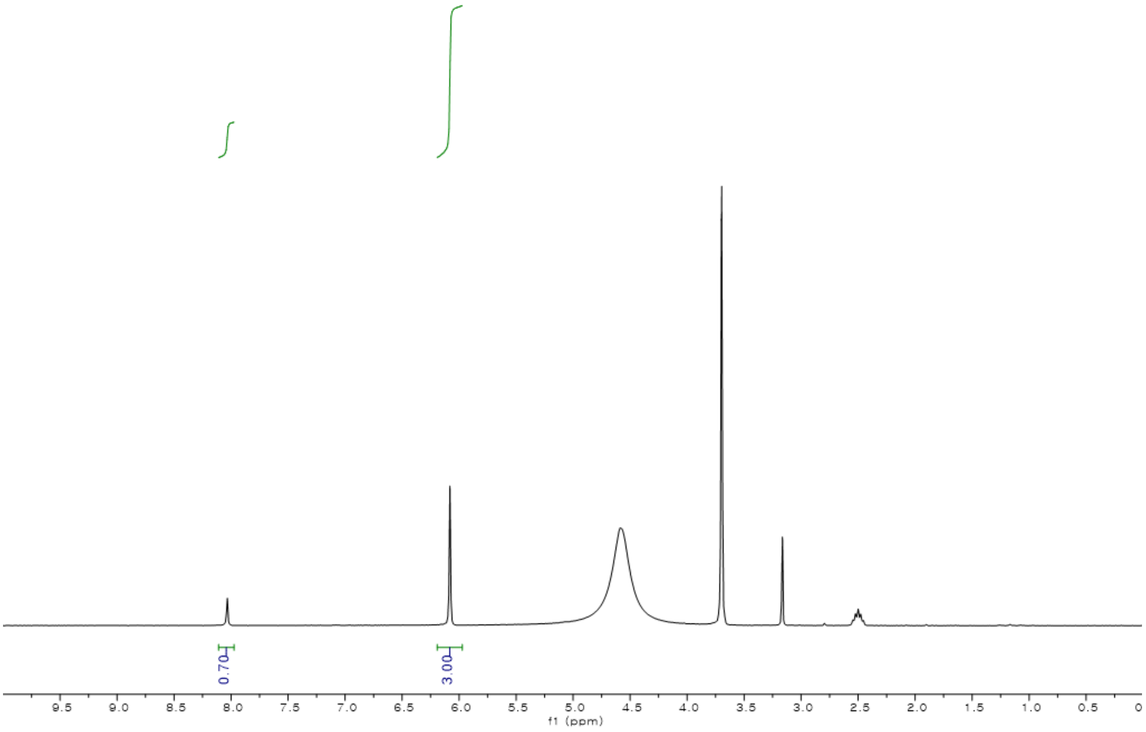

Peak integration with internal standard for treatment with Et_3N 0.4 M

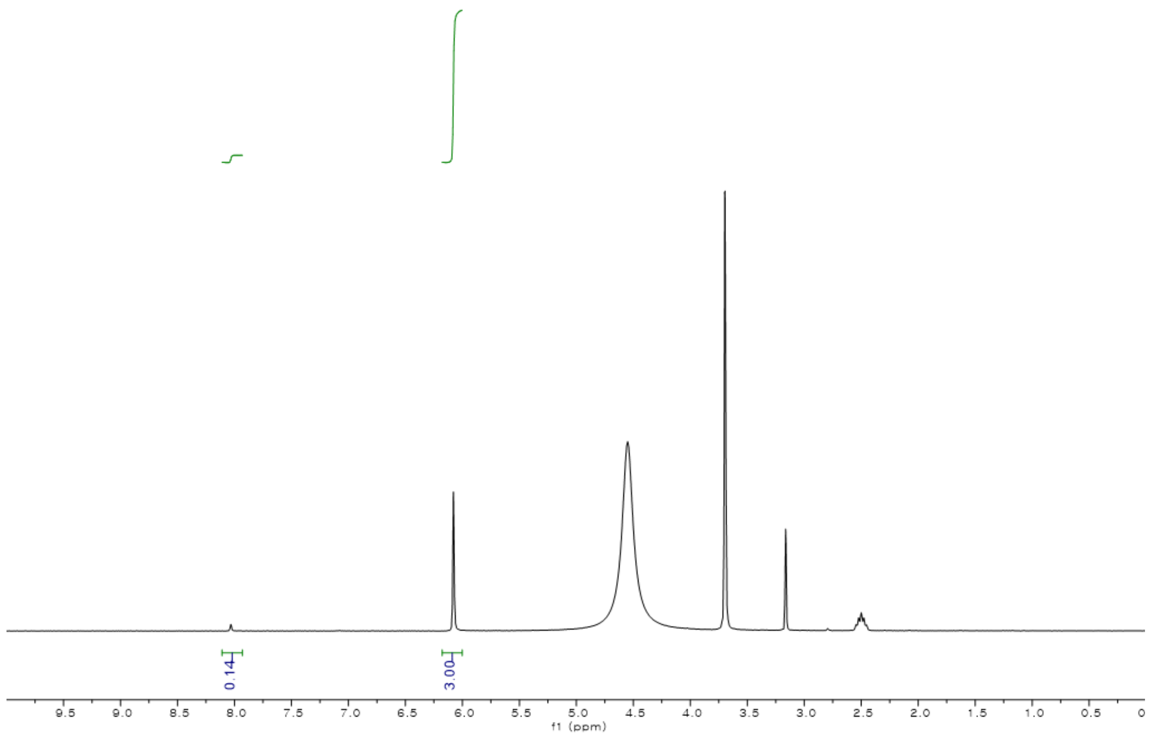

Peak integration with internal standard for treatment with Et_3N 0.5 M

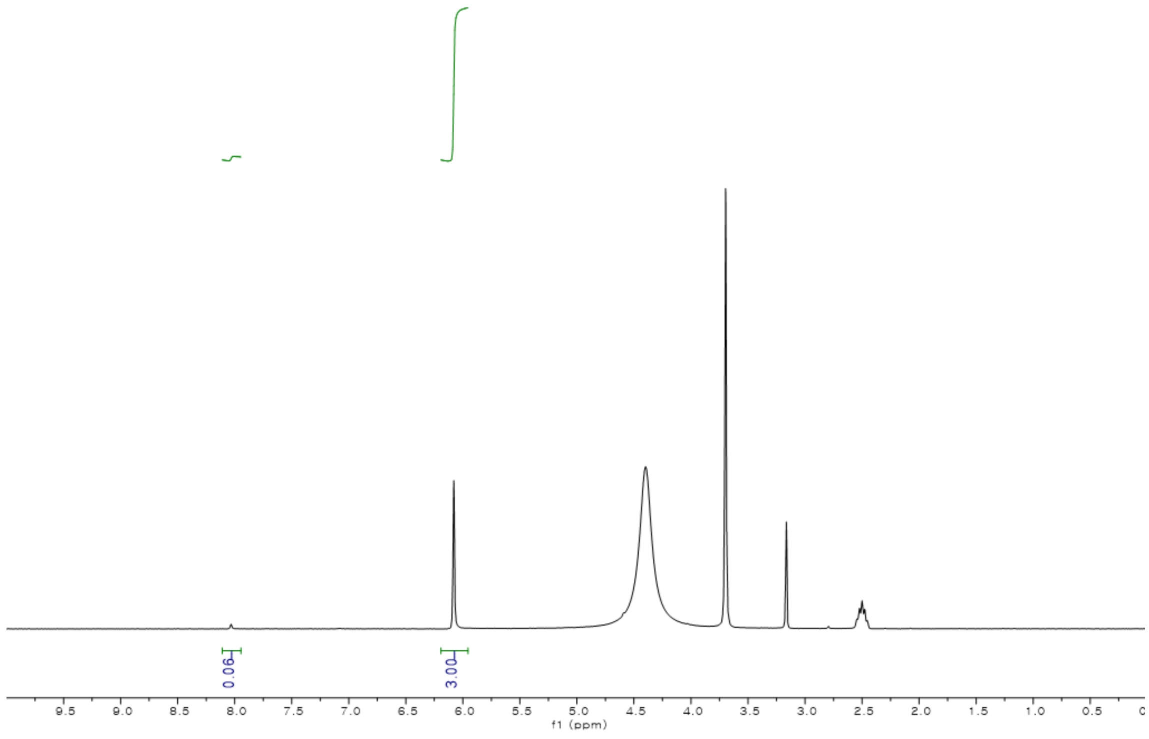

Peak integration with internal standard for treatment with aniline 0.1 M

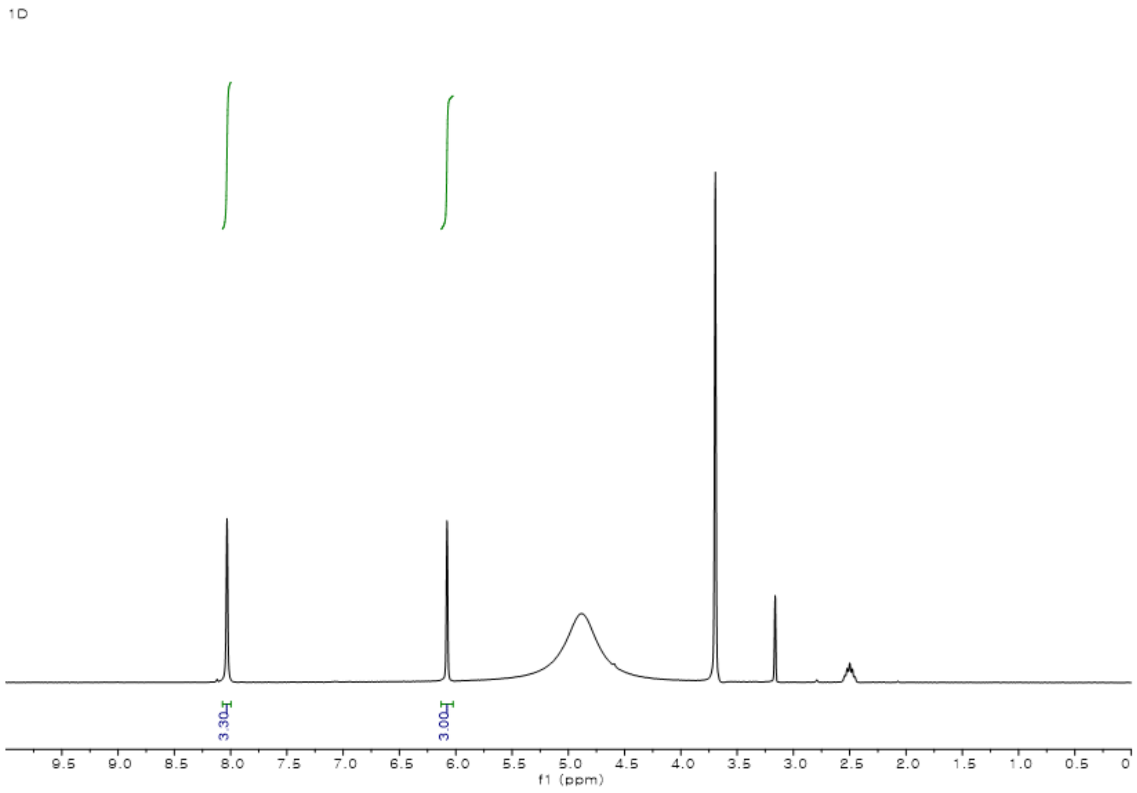

Peak integration with internal standard for treatment with aniline 0.4 M

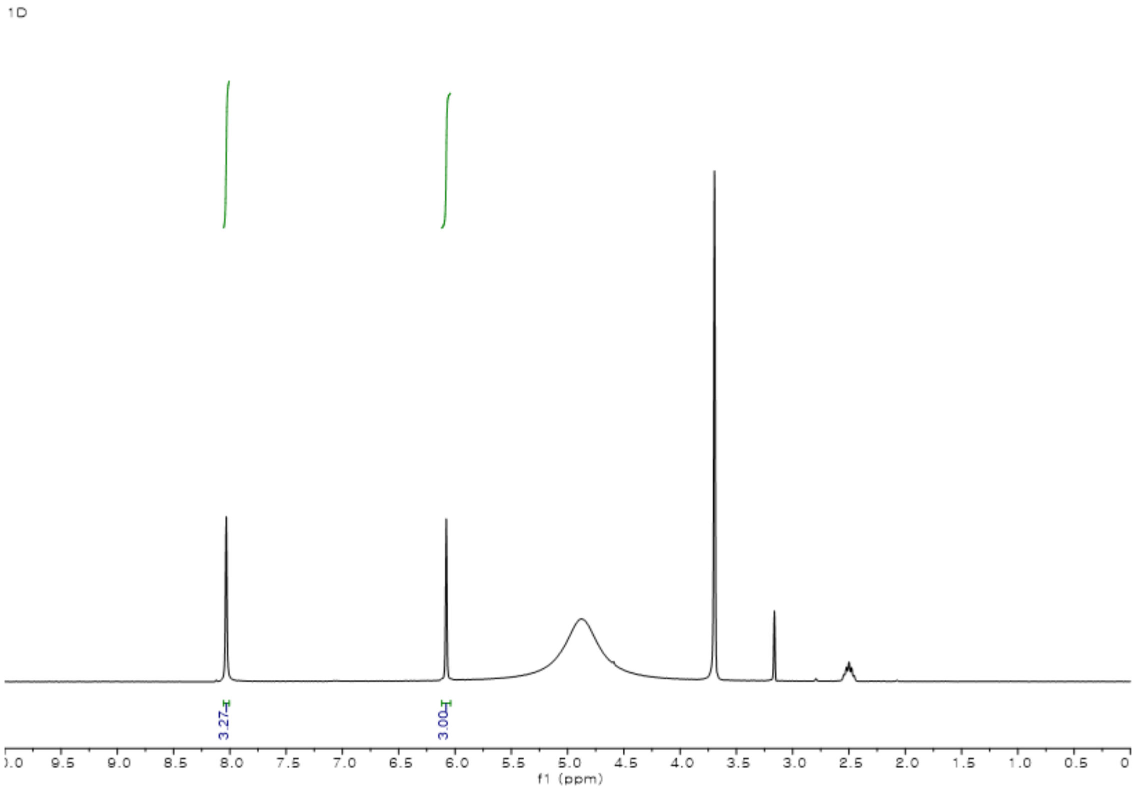

Peak integration with internal standard for treatment with 2,6-lutiine 0.1 M

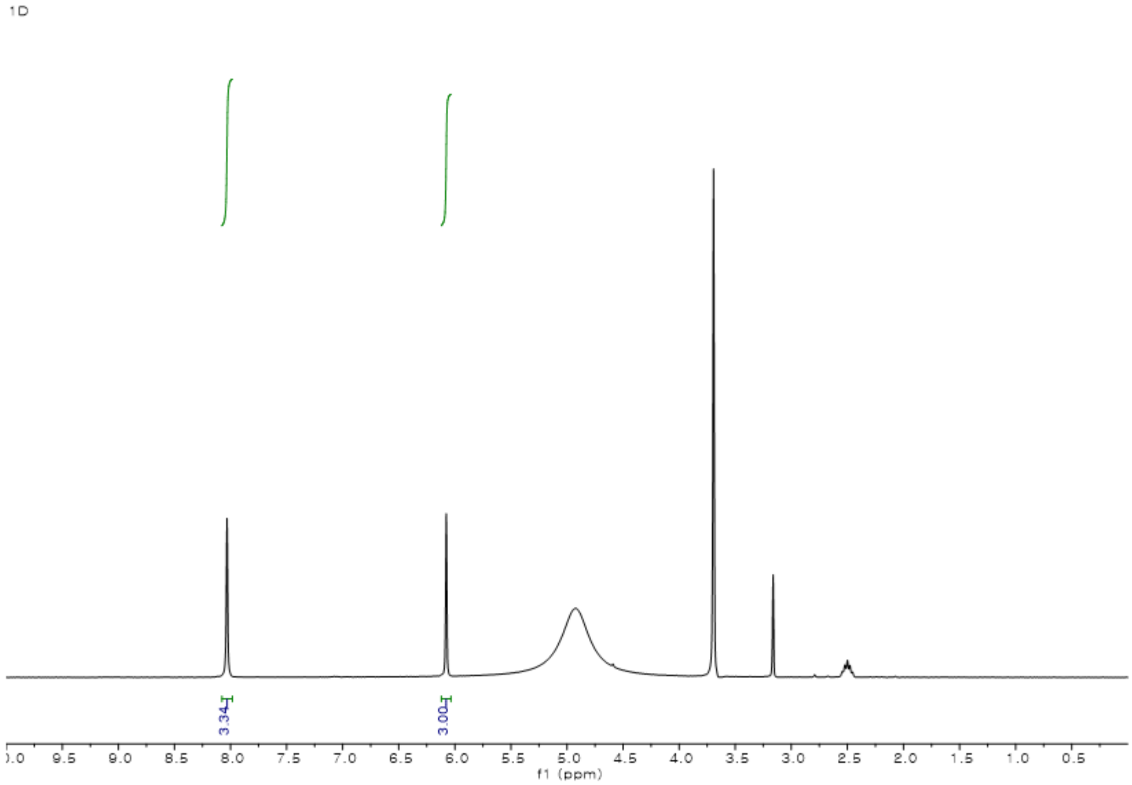

Peak integration with internal standard for treatment with aniline 0.5 M

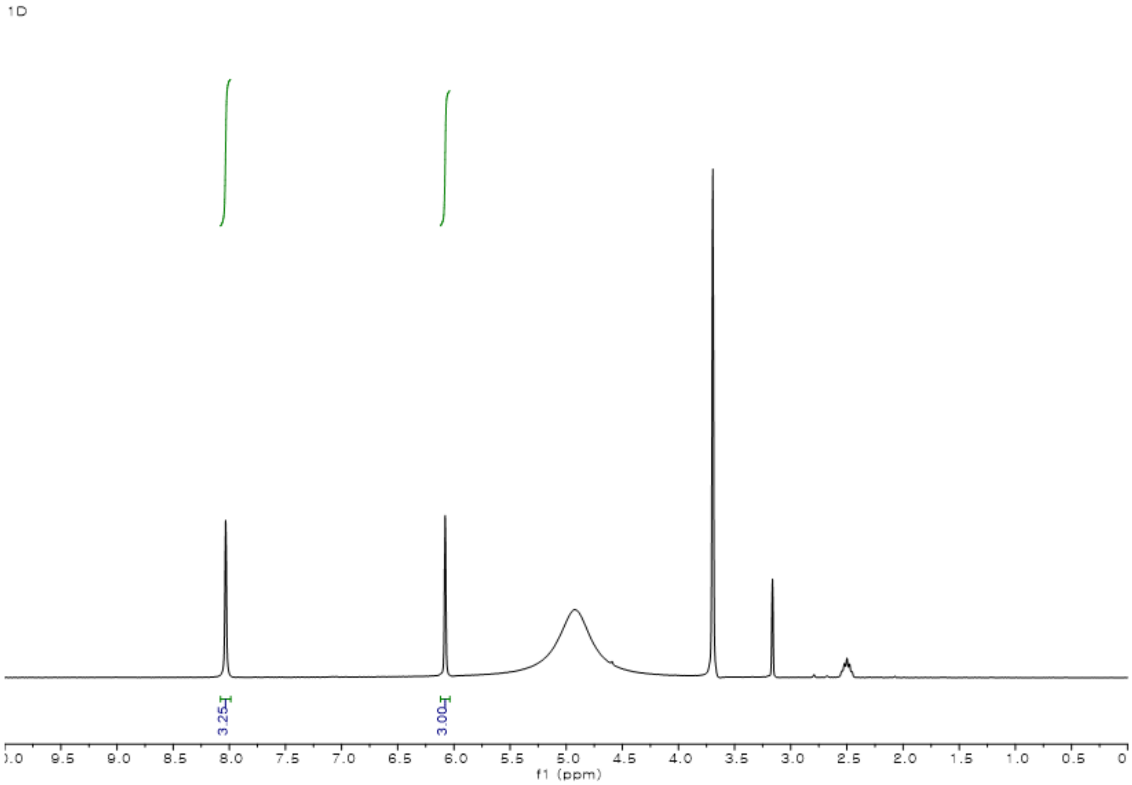

Peak integration with internal standard for treatment with benzylamine 5 M

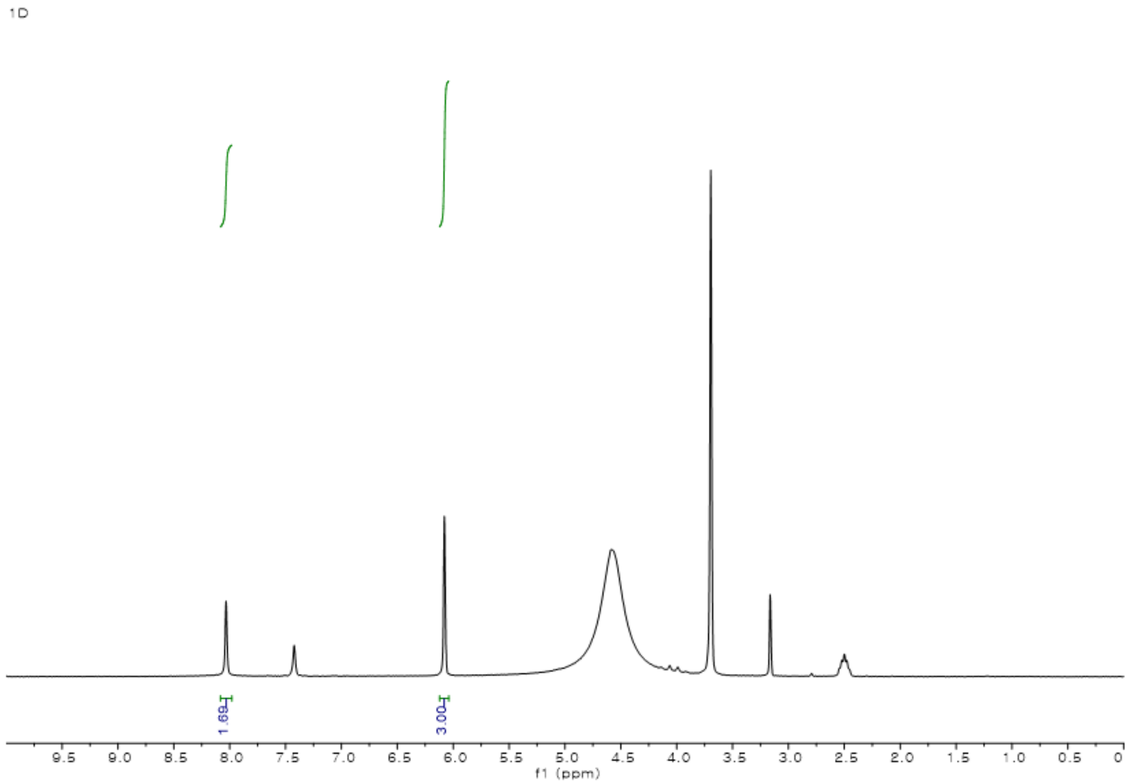

Peak integration with internal standard for treatment with benzylamine 6 M

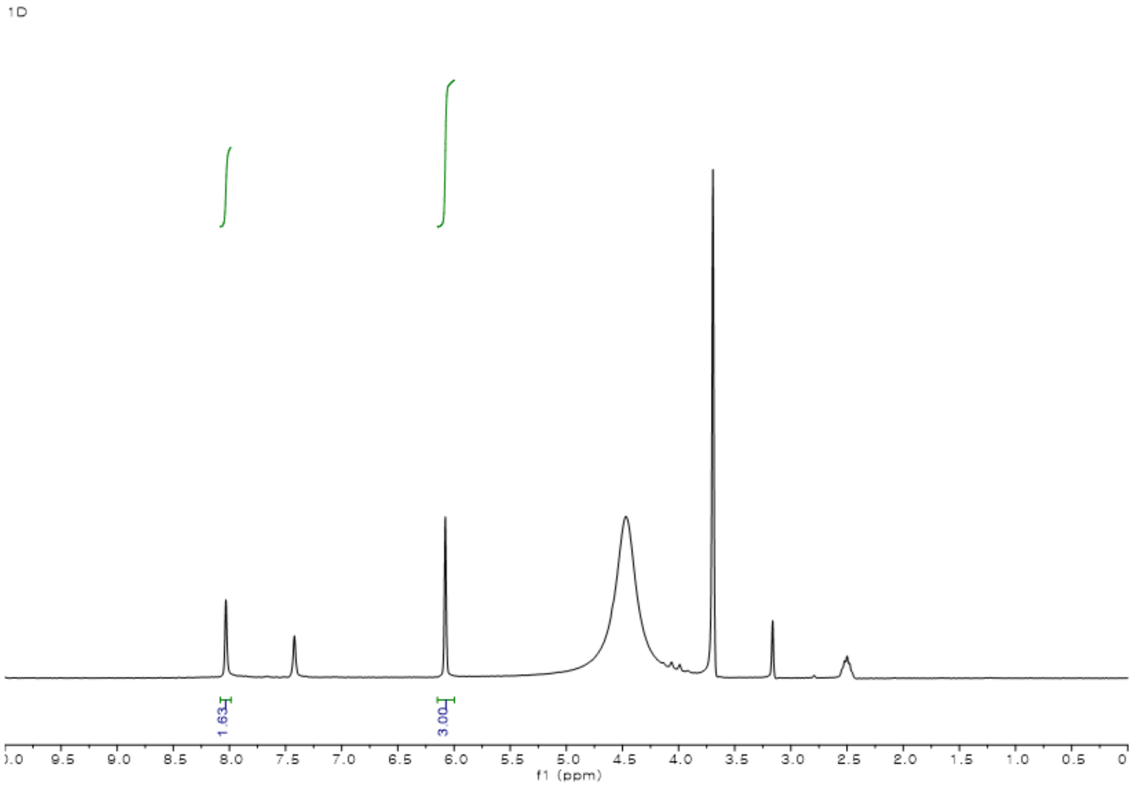

Supplement: Supplementary file 1 [file nanomaterials-14-00110-s001.zip › nanomaterials-2770839-supplementary.pdf]
